# From video to vital signs: a new method for contactless multichannel seismocardiography

**DOI:** 10.1038/s44325-024-00034-6

**Published:** 2025-01-10

**Authors:** Mohammad Muntasir Rahman, Bahram Kakavand, William Van Wurm, William L. Holman, Mohammad Reza Movahed, Amirtahà Taebi

**Affiliations:** 1 Biomedical Engineering Program, Mississippi State University, Mississippi State, MS USA; 2 Department of Pediatrics, Division of Pediatric Cardiology, Nemours Children’s Hospital, Orlando, FL USA; 3 College of Medicine, University of Central Florida, Orlando, FL USA; 4 Physician Assistant Studies Program, Mississippi State University, Meridian, MS USA; 5 Department of Surgery, Division of Cardiothoracic Surgery, University of Alabama at Birmingham, Birmingham, AL USA; 6 Sarver Heart Center, University of Arizona, Tucson, AZ USA; 7 College of Medicine, University of Arizona, Phoenix, AZ USA

**Keywords:** Cardiology, Health care

## Abstract

Seismocardiography (SCG) is a technique that non-invasively measures the chest wall’s local vibrations caused by the heart’s mechanical activity. Traditionally, SCG signals have been recorded using accelerometers placed at a single location on the chest wall. This study presents an innovative, cost-effective SCG method that utilizes standard smartphone videos to capture data from multiple chest locations. The analysis of vibrations from multiple points can offer a more thorough understanding of the heart’s mechanical activity compared to signals obtained solely from a single chest location. Our approach employs computer vision and deep learning techniques to extract and improve the resolution of multichannel SCG maps obtained by video capture of chest movement. We attached a grid of patterned stickers to the chest surface and recorded videos of chest movements during different respiratory phases. Using a deep learning-based object detector and a template tracking method, we tracked the stickers across video frames and extracted the corresponding SCG signals from sticker displacements. We also developed a robust algorithm to estimate heart rate (HR) from these chest videos and identify the optimal chest location for HR estimation. The method was tested on 28 chest videos captured from 14 healthy participants. The results demonstrated that our method effectively extracted multichannel SCG maps and enhanced their resolution with a mean squared error of 0.1078 and 0.0418 for right-to-left and head-to-foot SCG signals, respectively. We observed intersubject chest vibration patterns corresponding to cardiac events including opening and closure of the heart valves. Moreover, our algorithm accurately estimated HR from 1968 SCG signals extracted from the videos compared to the gold-standard HR measured from each subject’s electrocardiogram (bias ± 1.96 SD = 0.04 ± 2.14 bpm; *r* = 0.99, *p* < 0.001). The findings from this study underscore the potential of our approach in developing a cardiac monitoring tool using a smartphone that would be widely accessible to the general public and might provide more timely detection of diseases.

## Introduction

Cardiovascular diseases (CVDs) remain a significant global health challenge, causing an estimated 17.9 million fatalities annually^1^. This alarming figure represents roughly 32% of all deaths globally, highlighting the pressing need for efficient monitoring and diagnostic tools. The American Heart Association has emphasized the significant health and economic impact of CVDs, both in the United States and internationally^2^. Early detection and accurate diagnosis of heart conditions are crucial components of addressing this burden, as they can play an important role in enhancing patient outcomes and decreasing the CVDs’ strain on healthcare systems. Currently, a variety of techniques, both invasive and non-invasive, are employed for cardiac monitoring. Invasive methods are typically performed in clinical settings, whereas remote monitoring systems predominantly utilize non-invasive approaches, which are operable beyond the boundaries of healthcare facilities.

Over the past few years, seismocardiography (SCG) has appeared as a promising cardiovascular monitoring method, providing complementary information to well-established techniques such as electrocardiography (ECG)^3–6^. SCG is a noninvasive method that captures and quantifies subtle vibrations of the chest wall caused by the mechanical activities of the heart, including the opening and closing of cardiac valves, along with the rapid ejection and refilling of the ventricles. The SCG signal offers crucial insights into the timing of the aortic and mitral valves’ opening and closing, making it a powerful tool for investigating cardiac function^7–9^. Moreover, SCG has demonstrated potential in monitoring various cardiovascular problems, such as coronary heart disease, ischemia, aortic stenosis, myocardial infarction, and hemorrhage^10–16^.

Conventionally, SCG signals are measured from a single point on the chest using an accelerometer affixed to that spot. However, multichannel SCG measurements from various chest locations offer more precise and additional clinical data^17–20^. Current methods for capturing SCG signals from multiple chest points are primarily restricted to accelerometer arrays, and contactless techniques based on laser Doppler vibrometry and airborne ultrasound^5,21,22^. Accelerometer arrays have the disadvantage of requiring direct contact with the patient and are laborious for use in practice or remote monitoring. In addition, even ultra-lightweight accelerometers can cause non-negligible loading effects^22^. On the other hand, the current contactless laser or ultrasound-based methods are either expensive, challenging for inexperienced users, or require cumbersome equipment, hindering their application outside of clinical or research environments. What is lacking, therefore, is a convenient way of high-resolution multichannel SCG acquisition. To address this gap, the objective of this study is to introduce a novel approach using computer vision and deep learning to generate multichannel SCG maps from video footage of the human chest.

With the advancement of computer vision and sensors in recent years, vision-based techniques have gained popularity in extracting object motions from videos^23,24^. Recently, we developed a novel pipeline for measuring 0–30 Hz SCG signals from chest videos recorded by an ordinary smartphone^25^. The pipeline’s accuracy was validated by comparing vision-based signals with the gold-standard signals from industry-grade accelerometers. Results demonstrated a high accuracy in extracting SCG signals from chest videos, with all similarity indices exceeding 0.94^25^. The current study introduces several key advancements that extend the capabilities of our earlier work, providing both higher resolution and greater clinical relevance. First, unlike the earlier approach, which relied on a single-channel SCG signal, the current study utilizes a multichannel SCG approach. This is a significant leap forward in sensing chest vibrations, as it not only enhances the spatial resolution of the extracted signals but also enables precise identification of optimal chest locations for accurate physiological measurements, such as heart rate (HR). This multichannel capability allows for the construction of SCG maps that provide a more comprehensive view of cardiac activities, including rapid ejection phases and valve dynamics, which were not as detailed in our previous work.

Our approach involves tracking the motion of a grid of patterned stickers on the chest skin from chest videos recorded during different respiratory phases, i.e., breath-hold at the end of both inhalation and exhalation. For this purpose, we employ a deep learning-based object detector to identify and localize the stickers in the first frame of the videos, followed by template tracking to monitor each sticker’s movement across subsequent frames. This method enables us to extract SCG signals corresponding to the vibrations of each sticker. Furthermore, to enhance the resolution of the extracted multichannel SCG maps and provide a more detailed view of the chest vibrations, we propose a deep-learning model to interpolate between the measured SCG signals. This interpolation significantly improves the spatial resolution of the multichannel SCG maps, offering a more granular analysis of the chest’s mechanical activities. Additionally, we present an innovative algorithm to detect HR from the multichannel SCG signals, offering a more robust and accurate measurement compared to single-channel vision-based approaches. The findings of this study provide strong support for developing a smartphone-based, portable, non-invasive, and cost-effective multichannel SCG monitoring tool that could increase access to cardiac monitoring and potentially reduce time-to-diagnosis and healthcare costs associated with CVDs.

## Results

### Study protocol

A total of 17 subjects were recruited in this study, all of whom had no previous instances of CVDs. While data from the first 14 subjects (4 females: {S04, S05, S13, S14}, age: 23.50 ± 5.16 years, height: 170.80 ± 9.35 cm, weight: 70.07 ± 13.97 kg, BMI: 23.93 ± 4.07 kg/m^2^) was used to develop the vision-based algorithm, data from the remaining subjects (1 female, age: 35.33 ± 21.39 years, height: 169.33 ± 9.00 cm, weight: 83.22 ± 50.52 kg, BMI: 28.08 ± 14.14 kg/m^2^) was only used for training and testing the deep learning methods. One of the participants (S08) disclosed a history of epilepsy and exhibited diminished strength on the right side of the body in contrast to the left side. The study protocol received approval from the institutional review board of Mississippi State University. All participants gave informed consent and filled out a brief health condition survey. Also, consent was obtained for the publication of the chest photographs and videos from one of the subjects.

**Fig. 1:**
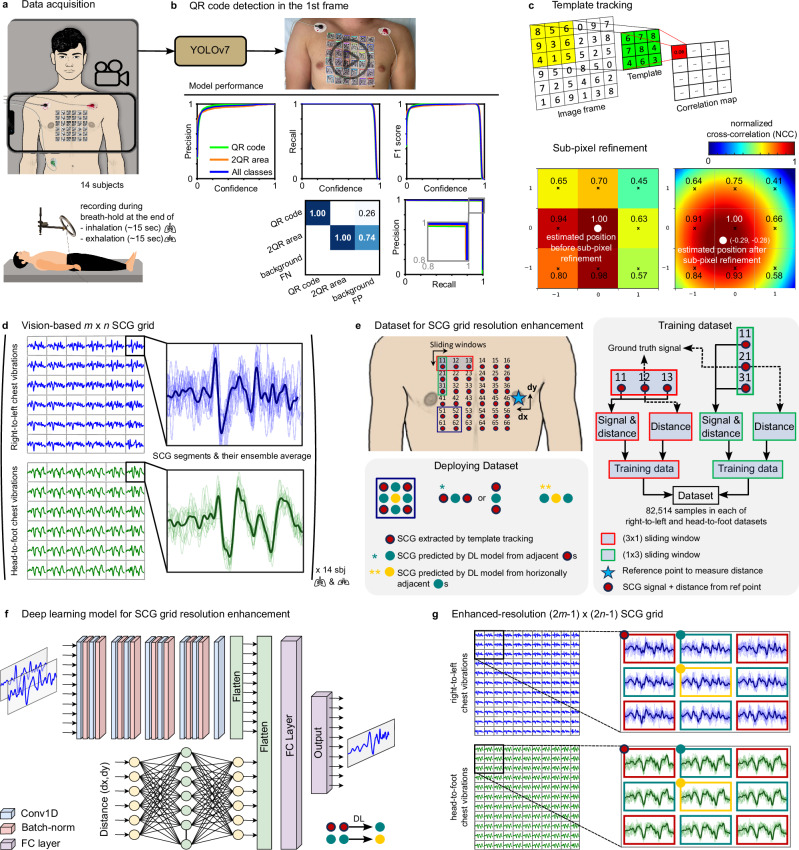
Vision-based multichannel seismocardiography (SCG) acquired by an ordinary smartphone camera. **a** Chest videos of 14 subjects were recorded during breath-hold maneuvers at end-inhalation and end-exhalation while a grid of patterned stickers was attached to their chest. **b** An object detection model was developed to determine the location of the patterned stickers in the first video frame. Consent was obtained for publication of the subject’s chest photograph. **c** Target tracking and sub-pixel refinement were used to determine the displacement of the sticker grid frame by frame. SCG signals in the right-to-left and head-to-foot directions were then calculated from these displacement signals. **d** Examples of *m* × *n* (=6 × 6) SCG grids. The right-to-left and head-to-foot SCG signals were plotted in blue and green colors, respectively. The magnified inset shows the SCG segments corresponding to cardiac cycles and their ensemble average (the darker segment). **e** To enhance the resolution of the multichannel SCG, two deep learning models were developed: one to interpolate the signals between two adjacent horizontal SCG signals (an example of the training sample for this model is shown in the red box), and the second one to interpolate the signals between two adjacent vertical SCG signals (a training sample is shown in the green box). **f** The architecture of the model for SCG grid resolution enhancement. **g** Examples of enhanced-resolution (2*m*−1) × (2*n*−1) SCG grids. The signals in the green boxes were predicted from the adjacent red boxes (either horizontal or vertical). The signals in the yellow boxes were predicted from the two adjacent horizontal green boxes.

To minimize movement artifacts during data collection, all subjects rested supine on a bed and were instructed to avoid any unnecessary body movements. We attached a *m* × *n* grid of stickers patterned with QR codes to the chests of the subjects to create high-contrast and trackable regions of interest. Modesty coverings were made available to all subjects, irrespective of sex, and two participants (S04 and S05) opted to use them during the data acquisition. For these two subjects, stickers were not applied to the areas covered by the modesty garments. The data acquisition system consisted of a smartphone (iPhone 13 Pro, Apple Inc, Cupertino, CA) capturing chest video at 60 frames per second (fps) and 3840 × 2160 pixel resolution. To minimize phone vibrations, a holder was employed to maintain the smartphone in a stationary position with the back camera directed at the subject’s chest (Fig. 1a), and a Bluetooth remote control initiated and stopped recordings. Simultaneously, single-lead ECG data was acquired using a data acquisition system (416, iWorx Systems, Inc., Dover, NH). A microphone connected to both the data acquisition system and smartphone provided audio timestamps at the beginning and end of each recording. These timestamps were used during the data analysis to synchronize the video and ECG data. Data was recorded during two 15-s breath-hold maneuvers: one at end-inhalation, the other at end-exhalation.

### Vision-based multichannel SCG

The objective of this study was to leverage computer vision techniques to extract a multichannel SCG map from chest video recordings. To accomplish this, we initially used a deep learning object detector, specifically You Only Look Once v7 (YOLOv7)^26^, to identify the initial location of the stickers on the chest in the first frame of the video. The object detection model was trained on a custom dataset of QR codes, which included two classes: (1) QR codes and (2) the gap between two adjacent QR codes enclosed by the QR code borders. The second class was included to improve the performance of the object detector after we observed instances in which the model mistakenly identified these regions as QR codes. After the training phase, the model’s performance was evaluated on the first frame of the videos recorded from the first 14 subjects under both breath-holding conditions as well as a third video captured from each subject (these third videos were not used in the rest of this study). These frames, unseen by the model during training, resulted in a total of 42 images with 2684 labels. The overall performance of the model is presented in Table 1 and Fig. 1b. Precision and recall values in Table 1 are calculated using the best confidence score of 85.4%. The high mean average precision score indicated the high precision of the model in detecting stickers, demonstrating the effectiveness of the training process. The confusion matrix in Fig. 1b is generated using a minimum confidence score of 25% and a minimum IoU threshold of 45%, which indicates that the model had a perfect score of 1.00 for correctly identifying both classes. Furthermore, the last row of the confusion matrix, i.e., the background FN, shows that the model did not miss any objects in either of the two classes to consider them as background objects. However, there were instances of false positives where the model misclassified certain background objects, such as the ECG electrodes and nipples, that did not belong to either of the target classes. We added a filter to our algorithm to remove these false positives. During testing, the model took 11.2 ms for inference, 1.5 ms for non-maximum suppression, and a total of 12.7 ms per 640 × 640 image at a batch size of 32.

**Table 1:** Object detection model performance is evaluated using precision, recall, and mean average precision (mAP)

Classes	Precision (%)	Recall (%)	mAP@.5 (%)	mAP@.5:.95 (%)
QR code	100	100	99.6	81.5
2QR area^a^	99.4	99.9	99.6	80.5
All (mAP)	99.7	99.9	99.6	81.0

Precision and recall are calculated based on the best confidence score, which is 85.4%. The mAP@.5 is computed at an Intersection over Union (IoU) threshold of 0.5. The mAP@.5:.95 shows the mAP calculated over a range of IoU thresholds from 0.5 to 0.95, with a step size of 0.05.

^a^2QR area: area between two adjacent QR codes enclosed by the QR code borders.

**Fig. 2:**
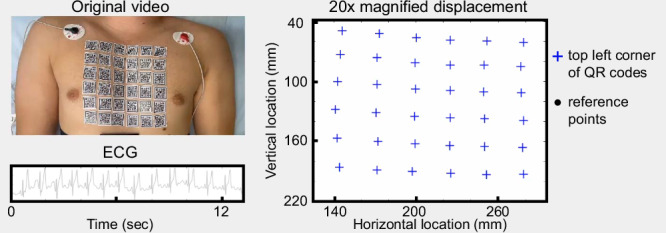
Sticker displacement visualization. The left panel shows a screenshot of the original video and the electrocardiogram (ECG) signal recorded from one of the male subjects (S03). The right panel shows the displacement of the stickers extracted using template tracking and subpixel refinement. Fixed reference points are included and the displacements are amplified 20 times for better visualization. In the PDF version of this article, please click anywhere on the figure or caption to play the video in a separate window. Consent was obtained for publication of the subject’s chest photograph.

Upon identifying the sticker locations in the first frame of each video, we utilized a template tracking algorithm to track their movements across the subsequent video frames^27^. Our template tracking algorithm provided the sticker positions at the pixel level as integer values. However, precise vibration measurement needed location information at the sub-pixel level. Therefore, a sub-pixel localization refinement technique was applied to the output of the template tracking algorithm (Fig. 1c)^28^. Figure 2 displays the extracted sticker motions for one of the male subjects. To further aid in the visualization of these subtle displacements, they were magnified with an amplification factor of 20. Subsequently, the SCG signals associated with every sticker location were calculated from the displacement signals. These SCG signals were then normalized and segmented into cardiac cycles using ECG R peaks determined by the Pan–Tompkin algorithm^29,30^. Figure 1d shows the acquired 6 × 6 grid of SCG signals after pre-processing for subject 3 during breath-hold at the end of exhalation. The blue and green waveforms display the SCG signals in the right-to-left and head-to-foot directions, respectively. In these figures, the ensemble average SCG signals are shown in darker blue and green colors. These results qualitatively demonstrate that SCG signals differ depending on where they are measured on the chest surface. Multichannel SCG grids for other subjects showed similar SCG variations (see Supplementary 1–14).

### SCG map resolution enhancement

The extracted SCG grids can provide a general overview of the cardiac activity. However, a higher-resolution grid can offer more detailed insights, particularly to analyze SCG spatial variations and propagation across the chest. Thus, we developed a deep learning convolutional neural network (CNN) to spatially interpolate the chest vibrations, enhancing the resolution of the SCG grids from *m* × *n* to (2*m*−1) × (2*n*−1). The architecture of the CNN model is shown in Fig. 1f.

We separately trained two versions of this model for interpolating the SCG signals in the right-to-left and head-to-foot directions. Data from the first 14 subjects were randomly split into training (90%) and validation (10%) sets. Data from the remaining three subjects (S15, S16, and S17) formed the test set to evaluate the model’s performance on unseen subjects. Table 2 presents the performance of the resolution enhancement models on both validation and test datasets in terms of mean squared error (MSE) and root mean squared error (RMSE). For the validation data, the models achieved an MSE and RMSE of 0.11 and 0.33, respectively, in the right-to-left direction, and an MSE and RMSE of 0.04 and 0.20, respectively, in the head-to-foot direction. The MSE and RMSE values were slightly higher for the test data. When combining data from S15, S16, and S17, the MSE and RMSE were 0.13 and 0.36 in the right-to-left direction, and 0.08 and 0.28 in the head-to-foot direction. Supplementary Fig. 15 illustrates sample predicted signals for the right-to-left and head-to-foot direction models (blue and green waveforms, respectively). These results showed that our proposed interpolation models accurately interpolated SCG signals in both right-to-left and head-to-foot directions.

**Table 2:** Performance of the resolution enhancement models on validation and test datasets

Data	Subject no.	Right-to-left		Head-to-foot	
		MSE	RMSE	MSE	RMSE
Val	–	0.11	0.33	0.04	0.20
Test	S15	0.14	0.38	0.07	0.27
	S16	0.14	0.37	0.08	0.28
	S17	0.07	0.27	0.08	0.29
	S15+S16+S17	0.13	0.36	0.08	0.28

Training and validation data were taken from the first 14 subjects, with a random split of 90% for training and 10% for validation. Test data was obtained from three new subjects (S15, S16, and S17) whose data were not included in the training phase. Mean Squared Error (MSE) and Root Mean Squared Error (RMSE) were used as performance metrics for the model in two directions: Right-to-Left and Head-to-Foot.

Subsequently, these models were employed to predict chest vibrations in the middle location of every pair of adjacent SCG signals, either horizontally or vertically oriented. Figure 1g presents an example of interpolated (2*m*−1) × (2*n*−1) grid of SCG signals in right-to-left and head-to-foot directions, where *m* = *n* = 6. The magnified inset displays a 3 × 3 portion of these grids with signals in red, green, and yellow boxes. The signals enclosed in the red boxes are the original SCG signals, extracted from the chest videos by the proposed template tracking and sub-pixel refinement. The signals in the green boxes are the predicted outputs, generated by the trained interpolation models using the adjacent horizontal and vertical pairs of the original SCG signals (in red boxes) as input. However, this interpolation pipeline alone was not enough to predict the SCG signals at the center of the four red-boxed original SCGs (i.e., yellow dots in Fig. 1e and yellow boxes in Fig. 1g). To fill these gaps, the same interpolation models were used to generate SCG signals from the horizontal pairs of the green-boxed signals.

**Fig. 3:**
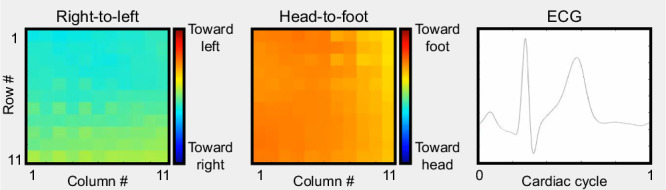
Time-varying right-to-left and head-to-foot chest vibrations of a male subject (S02) during one cardiac cycle. These maps were created using the ensemble average SCG signals in the enhanced-resolution (2*m*−1) × (2*n*−1) grids, where *m* = *n* = 6 for this subject. In the PDF version of this article, please click anywhere on the figure or caption to play the video in a separate window.

**Fig. 4:**
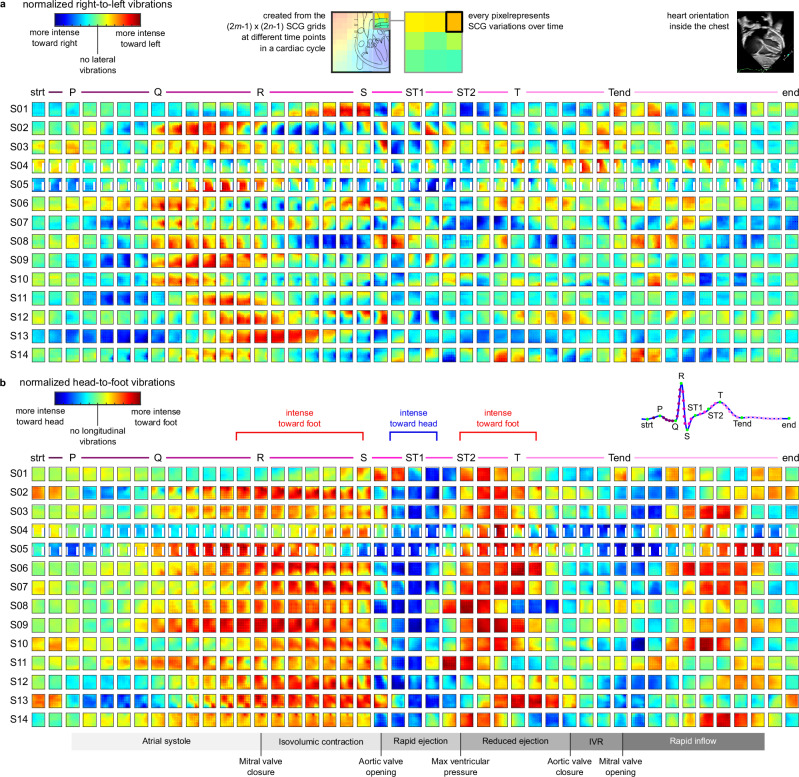
Enhanced resolution SCG maps at the end of exhalation. **a** Normalized right-to-left chest vibrations, and **b** Normalized head-to-foot chest vibrations for all 14 subjects during breath-hold at the end of exhalation. Each pixel of the maps shows the temporal variations of the corresponding ensemble average SCG signal during a cardiac cycle. Time-varying vibration maps were created using the enhanced-resolution (2*m*−1) × (2*n*−1) SCG grids and aligned with respect to the ECG reference points of each subject for better visualization of the results. It is noteworthy that the time step between the maps and the timing of maps corresponding to the same ECG reference point for different subjects are not uniform and depend on the cardiac cycle duration of each subject.

Leveraging the enhanced-resolution SCG grids, time-varying chest vibrations of the subjects were constructed using the ensemble average of the SCG signals at every chest location. Figure 3 shows the right-to-left and head-to-foot chest vibrations of Subject 2 for a cardiac cycle during breath-hold at the end of exhalation. For the right-to-left vibration maps, dark blue indicates more intense vibrations toward the right direction, while dark red signifies more intense vibrations toward the left direction. Similarly, for the head-to-foot vibration maps, dark blue represents more intense vibrations toward the head direction, and dark red corresponds to more intense vibrations toward the foot. Figure 4 shows selected frames of these maps for all 14 subjects at specific time points of a cardiac cycle with respect to the ECG fiducial points during breath-hold after exhalation. Visual inspection of these data reveals that predominantly blue and predominantly red maps appear to occur around the same point in the cardiac cycle with respect to the ECG reference across all subjects. This pattern suggests potential correlations with significant cardiac events such as the opening and closing of the cardiac valves, blood ejection, and ventricular filling. This observation not only raises questions about the underlying physiological sources of these vibrations for further investigation but also underscores the potential of our approach in providing novel insights into the mechanics of the heart. Similar patterns were observed in the vibration maps extracted from the data recorded during breath-hold after inhalation (see Supplementary Fig. 16).

Although our study trained the model from scratch to tailor it specifically to SCG signals, we recognize the potential benefits of fine-tuning. In future work, we plan to explore fine-tuning strategies to further enhance model performance. Additionally, conducting systematic ablation studies will allow a better understanding of the impact of different components within our model architecture.

### Vision-based heart-rate estimation

**Fig. 5:**
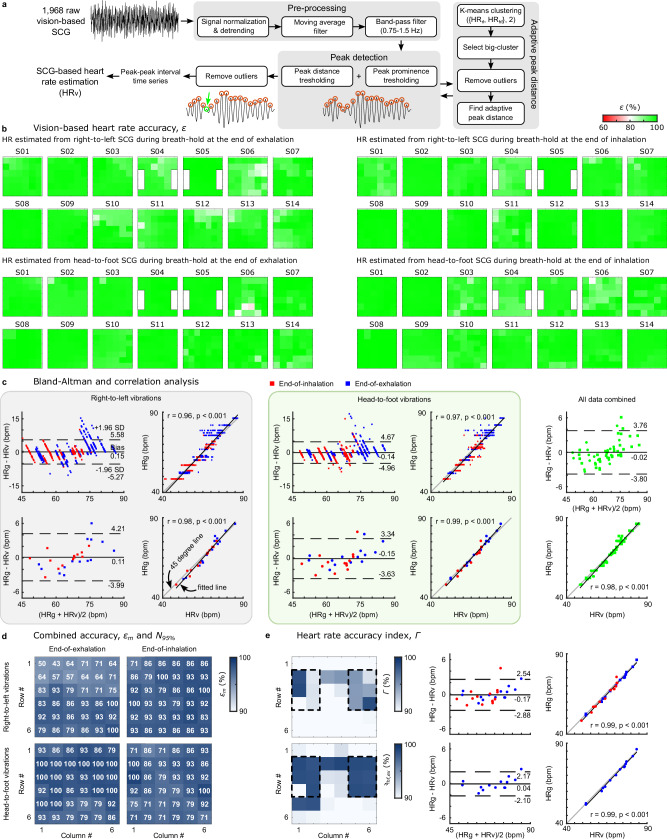
Heart rate (HR) estimation from the chest videos recorded by a smartphone camera. **a** An overview of the adaptive ECG-independent HR estimation algorithm to extract HR from the right-to-left and head-to-foot SCG signals. **b** Accuracy *ϵ* of the SCG-based HR compared to the gold-standard HR measured using ECG RR intervals. HR was not estimated from the white regions on the left and right side of the chest of Subjects 4 and 5 since these subjects chose to wear modesty coverings to cover these chest areas during the data acquisition. **c** Bland–Altman and Pearson correlation analysis. In the right-to-left and head-to-foot boxes, the plots on the top row are constructed using the HRs estimated from all chest locations, i.e., all nodes (QR codes) in the SCG grids, of all subjects. The plots on the bottom row were created using, an average accuracy for every subject. These average accuracy values were calculated for every subject based on the HR accuracy derived from all chest locations. The plots in the right panel (green data) were constructed utilizing the comprehensive dataset gathered from all participants' right-to-left and head-to-foot vibrations, collectively. **d** Combined accuracy, *ϵ*_*m*_ and *N*_95%_. The *N*_95%_ values are displayed in white font for each chest location, while the corresponding *ϵ*_*m*_ values are presented in a shade of blue. **e** HR accuracy indices, Γ and *γ*_*hf*,*ex*_. The regions enclosed with dashed lines show the chest locations for accurate estimation of HR using the proposed ECG-independent HR estimation algorithm and vision-based SCG signals. The Bland–Altman and correlation analysis on the right side illustrates the accuracy of the HR estimations in these regions.

We developed an adaptive ECG-independent algorithm for HR estimation from the vision-based SCG signals, *HR*_*v*_ (Fig. 5a). A total of 1968 different SCG signals were extracted from the chest videos of all 14 subjects. Instantaneous *HR*_*v*_ was calculated from all these signals in the right-to-left and head-to-foot SCG grids for every subject. Supplementary Video 1 shows the instantaneous *HR*_*v*_ calculated from the head-to-foot SCG signal extracted from one of the stickers for a sample subject (S03). The accuracy of the algorithm, *ϵ*, was determined by comparing the average of the SCG-based HR calculated from every chest location with the average gold-standard HR, *HR*_*g*_, obtained from ECG data as ${\epsilon }^{i,j}( \% )=(1-| H{R}_{v}^{i,j}-H{R}_{g}| /H{R}_{g})\times 100$, where *ϵ*^*i*,*j*^ is the accuracy of the *HR*_*v*_ calculated from the SCG signal at the *i*th row and *j*th column of the *m* × *n* SCG grid (where *i* ∈ {1, …, *m*} and *j* ∈ {1, …, *n*}). Figure 5b shows the *ϵ*^*i*,*j*^ estimated from the signals in the right-to-left and head-to-foot SCG grids. The more green represents the higher accuracy *ϵ*, the more red represents the lower accuracy, and the white color represents an accuracy of 80%. These color maps demonstrate that our adaptive algorithm accurately determined HR from most of the locations in the SCG grids. In most cases, *ϵ* was larger than 90% which is shown with different shades of green. The lowest accuracy was obtained from two adjacent locations in the head-to-foot SCG grid of Subject 6 recorded during breath-hold at the end of exhalation (*ϵ*^5,3^ = 78%, *ϵ*^5,4^ = 79.5%). Agreement between *HR*_*v*_ and *HR*_*g*_ was determined using Bland–Altman and Pearson correlation analysis. Figure 5c shows these results for HRs extracted from right-to-left and head-to-foot SCG grids. The top rows in the gray and green boxes illustrate the agreement of the HR estimations obtained from all the locations in the SCG grids (i.e., all stickers) with the corresponding *HR*_*g*_. The bottom row shows the agreement between the average *HR*_*v*_ for every subject ($=\mathop{\sum }\nolimits_{i = 1}^{m}\mathop{\sum }\nolimits_{j = 1}^{n}H{R}_{v}^{i,j}/(m\times n)$) and the *HR*_*g*_. These average *HR*_*v*_ values and their corresponding *HR*_*g*_ are also listed in Table 3. The bias or mean difference between the *HR*_*v*_ and *HR*_*g*_ ranged from −0.15 to 0.15 bpm. HRs estimated from the head-to-foot chest vibrations had the narrowest 95% confidence interval of ( − 3.63, 3.34) bpm. The correlation coefficient between *HR*_*v*_ and *HR*_*g*_ was 0.96–0.99 (*p* < 0.001) for different conditions. The Bland–Altman and correlation plots in the right panel of Fig. 5c were generated using all data, i.e., right-to-left and head-to-foot vibrations during breath-holds both after inhalation and exhalation. This collective analysis of the data also suggests that our ECG-independent HR estimation algorithm had high accuracy (bias ± 1.96 SD = −0.02 ± 3.78 bpm). Our results suggested that the HR estimated from the head-to-foot vibrations had a slightly higher agreement with the gold-standard HR compared to the estimations from the right-to-left SCG grids. In addition, while right-to-left vibrations underestimated the HR (bias > 0), the head-to-foot vibrations overestimated it (bias < 0).

**Table 3:** Gold-standard heart rate (HR_g_) and average vision-based heart rate (HR_v_) of the subjects during breath-hold after exhalation and inhalation

Subject No.	Breath-hold at the end of exhalation			Breath-hold at the end of inhalation		
	HR_g_ (bpm)	HR_v,rl_ (bpm)	HR_v,hf_ (bpm)	HR_g_ (bpm)	HR_v,rl_ (bpm)	HR_v,hf_ (bpm)
S01	51.86	52.28 ± 1.20	54.38 ± 0.72	57.00	55.29 ± 2.35	56.60 ± 0.43
S02	86.23	84.97 ± 0.58	83.33 ± 1.79	71.85	70.89 ± 0.13	71.27 ± 0.15
S03	62.94	64.57 ± 1.17	64.14 ± 1.03	62.12	63.44 ± 1.23	64.87 ± 1.90
S04	62.08	65.08 ± 2.86	62.53 ± 0.42	53.51	56.89 ± 0.86	56.53 ± 1.07
S05	75.16	75.53 ± 0.18	75.81 ± 0.19	68.08	67.79 ± 0.13	69.37 ± 1.48
S06	78.59	72.50 ± 3.80	74.45 ± 4.54	75.59	73.48 ± 1.85	70.98 ± 3.45
S07	66.88	67.47 ± 2.21	66.68 ± 1.27	60.09	61.98 ± 1.39	60.85 ± 2.06
S08	73.70	74.21 ± 0.17	73.54 ± 0.95	71.73	71.53 ± 0.38	71.54 ± 0.54
S09	70.14	70.78 ± 1.05	70.92 ± 0.34	68.16	68.07 ± 0.62	70.18 ± 1.06
S10	60.20	62.91 ± 2.55	62.30 ± 0.96	57.24	57.70 ± 0.45	58.70 ± 1.58
S11	52.59	54.51 ± 1.91	52.19 ± 0.41	48.40	48.46 ± 2.52	49.43 ± 0.70
S12	77.13	73.92 ± 3.52	76.62 ± 1.93	67.40	67.62 ± 0.73	68.33 ± 1.66
S13	81.90	79.10 ± 2.70	80.94 ± 2.14	68.14	67.80 ± 0.27	69.12 ± 1.13
S14	75.52	72.23 ± 2.56	74.29 ± 3.22	69.74	69.16 ± 1.88	70.24 ± 1.16

HR_v,rl_ and HR_v,hf_ (mean ± standard deviation) are calculated from the *m* × *n* SCG grids in the right-to-left and head-to-foot directions, respectively.

After assessing the accuracy of estimated HRs at different chest locations, this data was analyzed to identify the best inter-subject locations for accurate measurement of vision-based HR. First, we calculated the mean HR estimation accuracy ${\epsilon }_{m}^{i,j}$ for each location (*i*,*j*) across the data from all 14 subjects: ${\epsilon }_{m}^{i,j}={\sum }_{s}{\epsilon }^{i,j}/{N}_{s}$, where the index of summation *s* and *N*_*s*_ are the subjects and number of subjects, respectively. These results are presented in Fig. 5d maps. A dark blue refers to a higher *ϵ*_*m*_, while a white color shows an *ϵ*_*m*_ of 90%. Next, for each location, we calculated the percentage of the subjects whose HR estimation accuracy was larger than 95% (*N*_95%_). This was done separately for the SCG-based HRs derived from the vibration signals in right-to-left and head-to-foot directions and during breath-hold at the end of exhalation and inhalation, i.e., four different data sets. These *N*_95%_ values are reported as white numbers on Fig. 5d maps. Subsequently, an accuracy index *γ* was calculated for each location in every data set as ${\gamma }^{i,j}={\epsilon }_{m}^{i,j}\times {N}_{95 \% }^{i,j}/100$. To determine which data set leads to a more accurate HR estimation, we calculated an average accuracy index for every data set as $\overline{\gamma }=\mathop{\sum }\nolimits_{i = 1}^{m}\mathop{\sum }\nolimits_{j = 1}^{n}{\gamma }^{i,j}/(m\times n)$. The average index $\overline{\gamma }$ was larger for head-to-foot grids compared to the right-to-left grids: ${\overline{\gamma }}_{hf,ex}=90.66$%, ${\overline{\gamma }}_{hf,in}=82.09 \%$ vs. ${\overline{\gamma }}_{rl,ex}=75.81 \%$, ${\overline{\gamma }}_{rl,in}=87.37 \%$, where *γ*_*rl*,*ex*_ and *γ*_*hf*,*ex*_ denote the accuracy indices for the HRs derived from the right-to-left and head-to-foot SCG grids during breath-hold at the end of exhalation, and *γ*_*rl*,*in*_ and *γ*_*hf*,*in*_ are similar accuracy indices calculated from the data recorded during breath-hold at the end of inhalation. Finally, to determine the best inter-subject chest location for HR estimation, an accuracy index Γ^*i*,*j*^ was calculated for every chest location (*i*,*j*) by combining the indices calculated for the two head-to-foot data sets as ${{\rm{\Gamma }}}^{i,j}=({\gamma }_{hf,ex}^{i,j}+{\gamma }_{hf,in}^{i,j})/2$. Figure 5e shows the accuracy indices for the chest region of interest in this study. This map aids in identifying the optimal location on the chest surface for HR measurement using our proposed vision-based method. The areas highlighted in blue indicate regions of the highest accuracy. These results suggest that vision-based HR estimated from the chest regions enclosed by the midclavicular lines, sternal angle, and the fifth rib had the highest accuracy index (Γ > 90% and *γ*_*hf*,*ex*_ > 98%). These regions are shown by black dashed line in the Γ and *γ*_*hf*,*ex*_ maps of Fig. 5e. In this region, the estimated *HR*_*v*_ from head-to-foot vibrations during breath-hold at the end of inhalation and exhalation collectively had a bias of −0.17 bpm (1.96 SD: 2.71 bpm) and correlation coefficient of 0.99. Also, the estimated HR from the head-to-foot vibration at the end of exhalation had a bias ±1.96 SD of 0.04 ± 2.14 bpm.

### SCG spatial variations

**Fig. 6:**
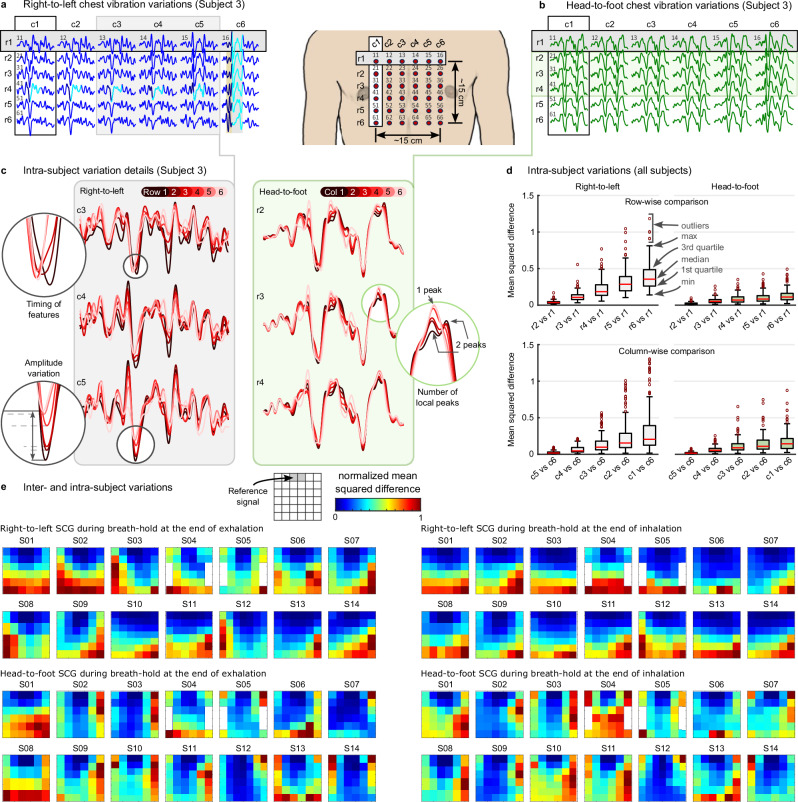
SCG spatial variation on the chest. **a**, **b** Ensemble averages of the right-to-left and head-to-foot SCG signals extracted from Subject 3's video recorded during breath-hold at the end of exhalation. **c** Examples of intra-subject variations include variations in the amplitude, timing, and number of the signal features, e.g., signal peaks. **d** (top panel) Mean squared difference between the signals in the second, third, fourth, fifth, and sixth (r2–r6) rows and the corresponding signals in the first row (r1) of the SCG grid generated using the data from all subjects, collectively; (bottom panel) Mean squared difference between the signals in the first, second, third, fourth, and fifth columns (c1–c5) and the corresponding signals in the sixth column (c6) of the SCG grid. **e** Normalized mean squared difference between the signals in the SCG grid and a referenced signal extracted from the sticker(s) on the manubrium, i.e., the top-middle sticker(s).

The established inter- and intra-subject variability of SCG signals have been extensively documented in previous studies^4,5,31^. Our innovative multichannel SCG acquisition method presents a practical and cost-efficient means to explore spatial variations in SCG. Illustrated in Fig. 6a, b are examples of SCG grids extracted from chest videos. The presented results showcase only the ensemble averages of the SCG segments. These findings portray the dynamic nature of SCG signals as measurement locations progress downward from the upper region of the chest. Focusing on the right-to-left vibrations, taking the sixth column (c6) as an example, the SCG peak located at the end of the darker segment of the signal shifted leftward when moving from the first row to the sixth row. Also, the SCG peak in the middle of the cyan segment in the initial row gradually shifted towards the end of the cyan segment by the sixth row. These two changes led to a wider valley between these two peaks in the cyan segment. Similar intra-subject variability was observed in the signals when the measurement location on the chest moved laterally. Other examples of intra-subject SCG variations are depicted in Fig. 6c, including variability in the timing, magnitude, and number of signal features in a particular period of the cardiac cycle. For example, in the green magnified inset, the signal in the first column exhibited two local maxima, whereas the signal in the sixth column had only a single maximum.

To evaluate the intra-subject variability across all subjects, we compared signals extracted from the second to the sixth rows (r2–r6) of the SCG grids with the signal from the first row (r1) of the corresponding column. The mean squared difference (MSD) of these signals is depicted in the top panel of Fig. 6d (row-wise comparison). The construction of this plot involved aggregating data from all subjects. For both the right-to-left and head-to-foot SCG grids, the MSD between r2 and r1 was the smallest. As one moved vertically from r2 to r6, the MSD progressively increased, with r6 compared to r1 exhibiting the highest MSD. These results highlighted a greater signal variability in the right-to-left vibrations compared to the head-to-foot vibrations. Specifically, for the right-to-left grids, the median MSD escalated from 0.03 (r2 vs r1) to 0.35 (r6 vs r1), while for the head-to-foot grids, it only shifted from 0.02 to 0.11. Moreover, in the right-to-left grids, the interquartile range expanded from 0.02 (r2 vs r1) to 0.22 (r6 vs r1), whereas in the head-to-foot grids, the increase was more modest, rising from 0.02 to 0.09.

Furthermore, a column-wise analysis was performed to evaluate signal variations as the measurement location moved farther from the heart, specifically toward the right side of the chest. This comparison involved calculating the MSD between the signals and their respective counterparts in the sixth column (c6). As illustrated in the lower panel of Fig. 6d, the results indicated a greater signal variability in the right-to-left SCG grids compared to the head-to-foot vibrations.

In addition to these row-wise and column-wise comparisons, each SCG grid’s signals were also compared with a reference SCG signal extracted from the sticker(s) situated at the top-middle, approximately positioned on the manubrium of the subjects. Figure 6e shows the normalized MSD (NMSD) between the SCG signals and the reference signal. Overall, the NMSD increased with the distance from the measurement location to the manubrium. Specifically, the NMSD showed a greater magnitude, i.e., higher signal variations, in the lower rows of the right-to-left grids. Conversely, in the head-to-foot grids, larger NMSD values were observed in the lateral columns. These findings also demonstrate the inter-subject variability of the SCG variations.

## Discussion

In this work, we established the feasibility of a novel multichannel SCG acquisition pipeline by extracting SCG signals from chest videos recorded by ordinary smartphones. This work builds on our earlier work in which we validated our method by comparing the vision-based SCG signals with the gold-standard signals recorded by industry-grade accelerometers attached to the chest skin^25^. The results of this work showed that right-to-left and head-to-foot SCG signals vary in a reproducible, cyclical nature during the cardiac cycle. Given the continuous, complex movement of the heart, SCG signals are influenced by various mechanical factors^4,5^. For instance, the movements of the heart are not only the events of the cardiac cycle such as the opening and closure of the cardiac valves, but also rotational movements caused by the shortening and twisting of the spirally arranged muscle fibers of the ventricles. Other thoracic vibrations may be caused by cardiovascular activities outside the heart, such as the filling and contraction of the aorta during systole and diastole. But what events in the cardiac cycle or blood flow within the chest most contribute to these chest surface vibrations? To interpret the SCG signals, we investigated their correlation with ECG signals and thus events of the cardiac cycle. Another key to interpreting the SCG events in this study is the relative magnitudes of the vibrations as detected by SCG in the lateral (i.e., right-to-left) and longitudinal (i.e., head-to-foot) directions.

The events of the cardiac cycle and their correlation with ECG waveforms were first described by Wiggers in the early twentieth century^32^. For this study, ECG tracings were simultaneously recorded with the chest videos. Thus, SCG signals obtained from the chest videos can be interpreted, in part, by their correlation with known events in the cardiac cycle as determined from corresponding ECG deflections. SCG, like echocardiogram, ECG, or other cardiac investigation techniques, may provide both intrapersonal information over time (such as information about the progression of heart failure in an individual patient)^13,33^ and interpersonal information that can be generalized across patients. For participants in this study, there were similar SCG patterns detected at the same time in the cardiac cycle across subjects. For example, a moderate lateral chest vibration was observed toward the end of the ECG PQ interval in most of the subjects (blue maps between P and Q in Fig. 4a and Supplementary Fig. 16a). These lateral vibrations are more intense during the breath-hold at the end of inhalation. To interpret these events, a brief review of the ECG timing and intervals is helpful. The cardiac cycle begins with the firing of the sinus node that results in the first ECG deflection known as the P wave. The P wave represents the right and left atrial contraction and ejection of the blood into the respective ventricles. The subsequent isoelectric PQ time interval provides enough time for the ventricles to fill with blood. Both actions, atrial contraction and blood flow, cause vibrations on the chest wall. The direction of the blood flow is at a shallow angle from top right to bottom left. This can be observed as the blue maps between P and Q in the lateral vibrations.

As another example, the head-to-foot SCG maps of the majority of the subjects in Fig. 4b had a large magnitude toward the foot direction (i.e., red color) during the ECG QRS complex. The QRS deflection depicts the electrical wavefront propagation that captures and depolarizes both ventricles. During this period, ventricles contract and generate pressure to close the mitral and tricuspid valves and open the aortic and pulmonary valves. This period is known as the isovolumic contraction (the ECG RS segment). Vibratory events during this segment involve muscle contraction and valve opening and closure.

The most consistent and dramatic example of similar intersubject findings was noted in the ECG cycle near the beginning of the ST segment, reaching a maximum intensity near the segment of the ECG known as the “J-point” (ST1 in Fig. 4). Near the ECG J-point, for all subjects, the magnitude of the SCG signal was greatest in the longitudinal orientation, moving towards the head (blue maps in Fig. 4b). This point in the cardiac cycle temporally relates to events including aortic valve opening and rapid ejection of blood from the left ventricle into the ascending aorta and from the right ventricle into the pulmonary artery. The direction of the blood flow is from the bottom left to top right at a steep angle. Also, ventricular pressure reaches their near-maximum values at the end of the ST segment. Another intersubject SCG consistency was noted during the rising inflection of the T wave and until the peak of the T wave. Events in this part of the cardiac cycle include reduced ejection into the aorta and pulmonary artery. It is noteworthy that the direction of blood flow undergoes a shift as it enters the descending aorta from the aortic arch, following its entry into the ascending aorta. This directional change contributes to the observable alteration in the head-to-foot chest vibrations between the S and T waves, as depicted in Fig. 4b, where the color transition from blue to red maps indicates this substantial variation.

In addition to intersubject consistency, SCG can provide subject-specific information, as demonstrated by Fig. 3 for a sample subject. In addition to the large amplitude vibration mentioned above that occurred for all subjects and can also be demonstrated in the video, the video provides additional information that likely corresponds to other key events in the cardiac cycle. Furthermore, SCG signals can provide insights into various cardiac events based on the orientation of the heart with respect to the SCG measurement locations and directions, i.e., right-to-left or head-to-foot. The heart lies diagonally in the thoracic cavity with the base pointing superiorly and posteriorly towards the right shoulder and the apex pointing downward, to the left, and slightly anteriorly. In the coronal view, the anatomical axis of the heart lies roughly 35 ± 10° relative to the horizontal axis of the body^34,35^. Thus, vibrations generated by mechanical activities of the heart that are aligned with the axis of the heart, such as the closure of the atrioventricular valves, would be expected to generate simultaneous longitudinal and lateral SCG amplitudes. In contrast, mechanical activities that are more vertically oriented, such as the closure of the aortic valve or flow into the aorta, would be expected to generate minimal lateral vibrations but rather primarily vertical vibrations. For example, at 14–15 s in Fig. 3, the large amplitude signal mentioned above that occurs at roughly the ECG R peak is likely sensed with movements toward the head and with concomitant rightward deflection in the lateral chest locations, as might be expected for vibrations proceeding along the anatomical coronal axis of the heart. This SCG pattern is likely caused by the closure of the atrioventricular valves, which is consistent with the information derived from both temporal relationship within the cardiac cycle as determined by ECG and the relatively large amplitude chest vibrations in the lateral and longitudinal directions. At 26–28 s, blood ejection into the ascending aorta and blood flow into the descending aorta generate a large amplitude signal in the head-to-foot SCG maps. Here, a large amplitude SCG component is measured toward the feet, while there are relatively low amplitude signals in the lateral directions, suggestive of anatomical structures more vertically oriented. These SCG patterns were also observed for other subjects and are shown with the mostly red maps in Fig. 4b around ST2-T location. These SCG fluctuations and those around the ECG J-point are the largest amplitude signals noted across subjects, which correspond to the opening of the aortic valve and rapid ejection phase. Another significant SCG finding in Fig. 3 occurs at 10–12 s, with both relatively large magnitude leftward and inferior vibrations noted towards the beginning of the ECG QR segment. Thus, the SCG likely captured the atrial contraction concluding with the mitral valve closure, which would be expected to proceed diagonally downward in a right-to-left pattern, and this is indeed demonstrated by the detected vibrations. These findings suggest that a multichannel SCG approach can provide information about cardiovascular events based on the orientation of different parts of the cardiovascular system.

The fact that aortic valve dynamics could be tracked based on the chest vibration maps in this study can open the door for developing a non-invasive method for diagnosing valvular heart conditions. For example, in patients with aortic regurgitation, aortic valve closure is delayed while in patients with aortic stenosis, the valve opening is delayed due to the narrowing or stenosis. Hence, further analysis of relationships between SCG and ECG may focus on the correlation between these vibration maps and valvular diseases. In addition, such analysis may allow serial monitoring of cardiac parameters such as QT intervals on the ECG, solely based on the proposed video-based method. Such information is potentially useful for monitoring the QT-prolonging effects of drugs that are prescribed in clinic. QT prolongation can be progressive over days and result in vulnerability to fatal arrhythmias. In addition, correlations between SCG, ECG, and echocardiographic data can potentially lead to SCG-based methods to monitor valve closure and valve insufficiency. Gallop rhythms due to third and fourth heart sounds are clues to worsening heart failure. Similarly, the appearance of SGR signals due to mitral or tricuspid valve regurgitation, whether by comparison to other patients or on serial SCGs from an individual patient, could be used together with other data (e.g., daily weights or pulmonary artery pressure measurements from an implanted CardioMEMS HF System^TM^) to optimize out-patient management. The non-invasive and low-cost features of the proposed SCG system are attractive for future developments, whether the system is used as a standalone method or in conjunction with other monitoring systems in the outpatient environment. Importantly, multichannel SCG maps would be analyzed by the patient’s smartphone so that unprocessed chest videos (large data files) would not require transmission from the patient’s smartphone to a central processing facility.

Our method demonstrated a robust correlation coefficient exceeding 0.98 with HR, positioning it as an attractive widely-accessible alternative for HR monitoring. This promising correlation suggests potential applications in diagnosing tachycardia-related issues, particularly in patients experiencing palpitations. However, this high correlation was observed in healthy subjects exhibiting sinus rhythm. In cases of patients with atrial fibrillation and varying myocardial contraction strength, it is conceivable that the HR correlation might be weaker. On the other hand, patients with supraventricular reentry tachycardia will have a consistent regular myocardial contraction that should lead to stable chest motion. This stability can be accurately detected and utilized to calculate HR reliably in such cases. The implications of our method may therefore vary depending on the cardiac conditions under consideration.

With a multichannel SCG system, it may be possible to evaluate left and right ventricular function. Deriving the heart rate and ventricular stroke volumes would be particularly helpful for non-invasively estimating cardiac output. Serial measurements of cardiac output that are made as medications are changed represent an important advance over current practice. Moreover, SCG recordings could diminish the frequency of outpatient encounters that physicians currently require to monitor the effects of changes to a patient’s therapeutic regimen.

While our current vision-based multichannel SCG method provides valuable insights into the patterns of the right-to-left and head-to-foot chest vibrations, it is unable to measure the dorsoventral component of the SCG signals, which is widely used and referred to in the literature^5^. This limitation arises from the fact that RGB video cameras can only capture 2D displacement information of the stickers in the chest plane. In the future, we plan to explore potential solutions to measure dorsoventral SCG using our method. In addition, the proposed vision-based method may be used to calculate gyrocardiograms (GCGs), i.e., the angular velocity of the thorax associated with cardiovascular activities, using the axial displacements in the right-to-left and head-to-foot directions. Future studies may evaluate the effectiveness of this method by comparing the vision-based GCG signals with those obtained by gold-standard sensors. Moreover, Fig. 4b demonstrated the intersubject similarity of SCG patterns in specific time periods of a cardiac cycle of an ECG waveform. Future studies may employ gold-standard imaging modalities, such as echocardiography, to pinpoint what exact cardiac events occur in these periods. This will also pave the way for the extraction of other important cardiac parameters and time intervals such as the stroke volume, pre-ejection period, left ventricular ejection time, and electrotechnical systole.

Finally, in this study, our method was tested on 17 healthy individuals, with data from 14 subjects used to develop the vision-based algorithm and data from the remaining subjects employed for training and testing deep learning methods. While this provides a solid foundation for evaluating the feasibility of our approach, the study was limited to individuals without previous CVDs, which restricts the immediate generalizability to broader populations, especially those with varying cardiac conditions. Furthermore, since our method employs a template tracking approach to determine displacement, its accuracy may be affected by the distance between the camera and the chest. Greater distances can reduce the resolution and detail of the stickers, leading to less precise tracking. The camera should ideally be positioned perpendicular to the chest surface, as an angled view can introduce interference between signals in different directions. Additionally, variations in lighting and smartphone models can influence the generalizability of our results. Future studies will be essential to validate the approach on more diverse populations in terms of age, body composition, and underlying cardiovascular conditions, as well as to investigate the impact of environmental factors on generalizability.

In summary, we presented a cost-effective method for multichannel SCG acquisition. Utilizing computer vision and deep learning, we developed a novel solution for extracting SCG grids from the chest videos recorded by ordinary smartphone cameras and found potential correlations between these grids and important cardiac events such as cardiac valve dynamics and blood flow ejection into the aorta. Furthermore, we developed and tested a deep-learning model to increase the resolution of the multichannel SCG grids, providing a more detailed view of the cardiovascular-induced chest vibrations. Our pipeline also included an innovative ECG-independent algorithm for heart rate detection from these vision-based SCG signals. The findings of this study highlighted the potential of our approach in creating an affordable and accessible cardiac monitor leveraging the wide availability of smartphones.

## Methods

### Multichannel SCG from chest videos

Target area detection: In this study, we affixed a grid of stickers patterned with QR codes to the chest surface of the subjects. The use of stickers was beneficial because it provided the intensity variation needed for the target tracking model to reliably track subtle chest motions^36^. These patterns provided distinct features to track throughout the video frames, facilitating the extraction of sticker displacements and SCG signals. The first step in our pipeline for capturing multichannel SCG involved the detection and localization of these stickers in the initial frame of the recorded videos. To accomplish this, we utilized YOLOv7^26^, a single-stage real-time deep learning-based object detection algorithm to process the first frame of the video and output the detected stickers along with their bounding boxes. YOLOv7 incorporates various techniques, such as extended efficient layer aggregation network, model scaling, and trainable bag-of-freebies, to enhance its performance compared to its predecessors. YOLOv7 was trained on QR codes using a custom-built labeled dataset comprising 10,713 images. This dataset was a combination of an online dataset^37^ and a dataset of annotated QR codes from chest videos of three additional subjects (S15, S16, and S17). Similar to the main 14 subjects, we also attached a grid of stickers to the chest of these subjects and videotaped their chest vibrations. This additional data ensured that the model learns from a diverse range of QR code images, including both publicly available data and specific samples captured during our data acquisition process. Since the stickers were placed close to each other on the chest in our experiments, there were instances when the object detection model detected the boundary regions of two adjacent stickers as a QR code. To avoid these misdetections, we defined an additional class by annotating the boundary regions of the two adjacent QR codes. Consequently, the model was trained on two classes: the QR codes and the boundary regions of two adjacent QR codes. For the training process, the model was initialized from a pre-trained model that was previously trained on the COCO dataset. The training was conducted for 100 epochs, completed in 7.14 h, using a batch size of 32 and an image size of 640 × 640 pixels. The Adam optimizer was used with an initial learning rate, momentum, and weight decay of 0.01, 0.937, and 0.0005, respectively.

Template matching: Once the initial locations of the stickers in the first frame of the video were identified by the object detector, template matching was employed to track the motion of each sticker across subsequent video frames. The algorithm begins by defining a template that captures the essential characteristics and appearance of the object. It then compares the reference template with different regions of an image or frames in a video sequence. The goal is to find the regions that most closely match the template, indicating the object’s presence and location. This is achieved by sliding the template over the image and determining the location with the highest similarity between the template and the overlapped region of the image. In this study, the templates were defined by the bounding boxes of the stickers in the first frame determined by the object detector.

Assume that a total of *K* stickers were attached to the subject’s chest and were defined as $q{r}_{k}=(({x}_{k}^{t},{y}_{k}^{t}),({w}_{k},{h}_{k}))$, where $({x}_{k}^{t},{y}_{k}^{t})$ was the top-left corner and (*w*_*k*_, *h*_*k*_) was the width and height of the *k*th sticker (*k* ∈ {1, …, *K*}). These *K* stickers were eventually mapped into a *m* × *n* grid based on their respective position on the chest. Now assume that all *K* stickers were correctly detected in the first frame, i.e., at *t* = 0, by the object detector. Let *T* = (*T*_1_, …, *T*_*K*_) be the set of *K* templates as defined by the bounding boxes of the stickers, with the size of *T*_*k*_ being *w*_*k*_ × *h*_*k*_. For each template, the template tracking algorithm then involved sliding a window, sized according to the template, over the entire video frame. It then calculated the similarity between the template and each region of the frame, identifying the region with the highest similarity index.

Searching for *K* stickers in a high-resolution image is computationally expensive, particularly when every template is slid over the entire image. Given that the videos were captured under controlled conditions with minimal chest movements, we assumed the stickers had only subtle displacements, mainly due to the cardiovascular-induced chest vibrations. Therefore, to reduce the computational cost, we defined a region, slightly larger than the template in the first frame, as the search region to locate the template in subsequent frames. Let *I*(**x**, *t*) be the video frame at time *t*, where **x** = (*x*, *y*) contained the pixel coordinates. Let assume *I*_*k*_(**x**, *t*) was the search region of size *W*_*k*_ × *H*_*k*_ for the corresponding template *T*_*k*_ at time *t*. For each template *T*_*k*_, we slid a window with the size of *T*_*k*_ over *I*_*k*_(**x**, *t*) and measured the similarity between *T*_*k*_ and the overlapped region on the *I*_*k*_(**x**, *t*). The similarity was determined using the normalized cross-correlation coefficient, *ρ*. By identifying the sliding window position with the highest similarity score, we found the best match for every template *T*_*k*_ in every video frame, indicating the object’s location in that video frame. This process enabled tracking the motion of every sticker throughout the video sequence.

SCG calculation: The template matching algorithm provides the object’s position with pixel-level precision. However, the exact position of the stickers in each frame may involve fractional pixel values. Therefore, a sub-pixel registration technique was used to further enhance the accuracy of our sticker tracking. For this purpose, a quadratic polynomial surface was fitted to the intensity values within a 3 × 3 pixel region centered around the pixel with the maximum *ρ* (Fig. 1c). It is worth noting that while using a larger region for curve fitting may yield more accurate estimations, it increases the computational cost. The quadratic surface equation resulting from the fitting of these nine points can be expressed as *f*(*x*, *y*) = *a*_0_ + *a*_1_*x* + *a*_2_*y* + *a*_3_*x*^2^ + *a*_4_*xy* + *a*_5_*y*^2^, where *a*_0_, …, *a*_5_ represent the six unknown constant coefficients of the quadratic surface. To estimate these constants, a pseudo-inverse computation was performed after substituting the nine points into the quadratic surface equation as

1 $$\left[\begin{array}{ccc}f(-1,1)&f(0,1)&f(1,1)\\ f(-1,0)&f(0,0)&f(1,0)\\ f(-1,-1)&f(0,-1)&f(1,-1)\end{array}\right]=\quad \left[\begin{array}{ccc}\rho ({x}_{max}-1,{y}_{max}+1)&\rho ({x}_{max},{y}_{max}+1)&\rho ({x}_{max}+1,{y}_{max}+1)\\ \rho ({x}_{max}-1,{y}_{max})&\rho ({x}_{max},{y}_{max})&\rho ({x}_{max}+1,{y}_{max})\\ \rho ({x}_{max}-1,{y}_{max}-1)&\rho ({x}_{max},{y}_{max}-1)&\rho ({x}_{max}+1,{y}_{max}-1)\end{array}\right]$$

where (*x*_*max*_, *y*_*max*_) are the coordinates of the maximum *ρ*. Once the coefficients *a*_0_, …, *a*_5_ were obtained, the extreme point of the quadratic fitting surface was determined using ∂*f*(*x*, *y*)/∂*x* = 0, and ∂*f*(*x*, *y*)/∂*y* = 0 as ${x}_{sub}=(2{a}_{1}{a}_{5}-{a}_{2}{a}_{4})/({a}_{4}^{2}-4{a}_{3}{a}_{5})$, and ${y}_{sub}=(2{a}_{2}{a}_{3}-{a}_{1}{a}_{4})/({a}_{4}^{2}-4{a}_{3}{a}_{5})$, where (*x*_*sub*_, *y*_*sub*_) represents the optimal sub-pixel position of the maximum *ρ*.

Upon applying the template matching algorithm and refining the sub-pixel localization for each frame, we obtained the sub-pixel coordinates of the upper-left corner of each sticker frame by frame. The relative displacement of each detected sticker between the current frame *I*(**x**, *t*) and the first frame *I*(**x**, *t*_0_) in right-to-left and head-to-foot directions were calculated as $d{x}_{k}^{t}={x}_{k}^{t}-{x}_{k}^{{t}_{0}}$, and $d{y}_{k}^{t}={y}_{k}^{t}-{y}_{k}^{{t}_{0}}$, where $d{x}_{k}^{t}$ and $d{y}_{k}^{t}$ represent the displacements in the right-to-left and head-to-foot directions, respectively, for the *k*th sticker. $({x}_{k}^{{t}_{0}},{y}_{k}^{{t}_{0}})$ is the coordinates of the *k*th sticker in frame *I*(**x**, *t*_0_), while $({x}_{k}^{t},{y}_{k}^{t})$ represents the coordinates of the same sticker in frame *I*(**x**, *t*). To calculate the acceleration (i.e., SCG signal) from the displacement signal, we used the central difference method, which provides a balance between accuracy and noise sensitivity. In this method, the derivative is calculated by considering the slope between neighboring data points on both sides. The velocity and acceleration signals were calculated as *v*(*t*) = [*d*(*t*+1)−*d*(*t*−1)]/2. Δ*t*, and *SCG*(*t*) = [*v*(*t*+1)−*v*(*t*−1)]/2. Δ*t*, where *d* and *v* represent the displacement and velocity, and *Δt* is the time step between data points.

Cardiac cycle segmentation: Each subject’s ECG was recorded simultaneously with the chest videos. While the vision-based SCG signals were captured at 60 fps, the ECG signals were recorded at a sampling frequency of 5000 Hz. Therefore, to align the SCG signals with the ECG signals, the signals were resampled to 5000 Hz using linear interpolation. This process also helped make a smoother SCG signal by reconstructing the missing features without losing information in either time or frequency. We then applied a band-pass filter (1–30 Hz) to the SCG signals. To segment the SCG signal into cardiac cycles, we followed a method similar to our previous work^25^. Specifically, we defined a window size based on the average heart rate calculated from the ECG signal. The window started 1/4 of the average heart rate before each R wave. After segmenting the SCG signals, we further resampled each segment to a fixed number of sample points (5000 points in this study). This consistent segment length across all subjects ensured uniform input sizes for our CNN model.

### SCG grid resolution enhancement using CNN

After extracting SCG from each of the *K* stickers, we mapped these signals onto a *m* × *n* grid, corresponding to their specific positions on the chest. Subsequently, we implemented a deep learning model composed of a 1D CNN and a multilayer perceptron (MLP) network to enhance SCG grid resolution to (2*m*−1) × (2*n*−1). The CNN model processed two SCG signals derived from the *m* × *n* grid as input, whereas the MLP network utilized the position information of stickers relative to a reference point (left nipple) as its input. Each of these models generated a feature vector. The two feature sets were then combined to predict the SCG at the midpoint between the two known signals. The architecture and data flow of the model is illustrated in Fig. 1e–g.

Network architecture: The CNN model had five convolutional blocks. The first four blocks contained two Conv1D layers, each followed by batch normalization and ReLU activation layers. The Conv1D layer applied 1D convolution operations to the input data, extracting relevant features and capturing spatial patterns. The batch normalization layer normalized the output of the Conv1D layer, aiding in stabilizing and accelerating the training process by reducing internal covariate shift^38^. The ReLU activation layer introduced non-linearity to the network. The kernel size, stride, and padding parameters for the first convolutional blocks were set to 5, 1, and 2, respectively. For the rest of the convolutional blocks, these parameters were set to 3, 1, and 1. The last convolutional block contained one Conv1D layer only followed by a ReLU activation layer and flattening. Additionally, an MLP network incorporated the lateral and longitudinal distance information of each sticker from a reference point. It consisted of two fully connected units, each comprising a linear transformation followed by a ReLU activation layer. The outputs of the CNN and MLP networks were concatenated and processed by a sequence of two fully connected layers. The first one applied a linear transformation to the concatenated input, followed by a ReLU activation layer, while the second layer was responsible for regressing and interpolating the output signal.

Dataset: After pre-processing and segmenting the data into cardiac cycles from all subjects, we created two datasets of SCG signals: one consisting of the right-to-left SCG signals and the other using the head-to-foot signals. The dataset was divided into training, validation, and testing sets. The training and validation data were created from the first 14 subjects and randomly split into 90% and 10%, respectively. The test data were taken from the remaining three subjects (S15, S16, and S17). Each sample in the datasets was constructed by considering SCG signals extracted from three adjacent stickers and their corresponding distance from a reference point (Fig. 1e). To generate these datasets, two sliding windows of size 1 × 3 and 3 × 1 were employed and successively slid horizontally and vertically across the *m* × *n* sticker grid, allowing for the collection of every three adjacent SCG signals and their lateral and longitudinal distance from the reference point. Within each window, the SCG signals from the first and third stickers, and the distance information of all three stickers formed a sample input for the training. The SCG signal from the middle sticker was designated as the ground truth for the predicted signal at the midpoint between the first and third stickers. To augment the dataset, we created three additional sets of SCG segments using the same length of 5000 sample points as described in the “Cardiac cycle segmentation” subsection, but starting from 2/4, 3/4, and the total of the average heart rate before each R wave, respectively. By repeating this generation process, we created a total of 82,514 samples in each dataset based on the signals recorded from the first 14 subjects during the two breath-holding conditions. For the test set, which was taken from the remaining three subjects, we used the same sliding window method but did not apply any data augmentation, resulting in a total of 1971 samples in each dataset.

Network training and deploying: To interpolate the SCG signals, we trained two separate models, one for each direction (right-to-left and head-to-foot), with a maximum of 300 epochs. To mitigate overfitting, we implemented early stopping to terminate training if the model’s performance no longer improves on the validation set for a predefined number of epochs. To learn effectively, the model used the Adam optimizer with a starting learning rate of 0.001. This rate automatically decreased if the validation performance stagnated for several epochs. This approach facilitated convergence to a superior solution while avoiding convergence to local minima. A batch size of 128 was utilized, and the input size for the CNN was set to 5000 × 2 (corresponding to the 2 SCG segments extracted from the first and third stickers in each sample), while the MLP network had an input size of 6 × 1 (corresponding to the lateral and longitudinal distances of the three stickers in each sample from the reference point).

The trained models were initially deployed to predict the SCG signal in the middle point (green circles in Fig. 1e) between each pair of horizontally or vertically adjacent stickers (red circles in Fig. 1e). However, during the first phase of deployment, these models were unable to predict the signals corresponding to the yellow circles in Fig. 1e. To predict the SCG signals at these locations, we utilized the same models, using the predicted signals from the horizontal direction as input to predict the signals at the yellow circle.

### Heart rate calculation from chest videos

Our HR estimation algorithm comprised two sequential steps, illustrated in Fig. 5.a. In the initial step, an approximate HR was estimated, while the subsequent step refined this estimation for greater accuracy. In the first step, each SCG signal in the right-to-left and head-to-foot *m* × *n* grids was first normalized and detrended. To smooth the signals and eliminate short-term fluctuations or noise, a moving average filter with a window size of 0.6 × *f*_*s*_ was applied, where *f*_*s*_ represents the sampling frequency. Subsequently, a 5th-order Butterworth bandpass filter (0.75–1.5 Hz) was used to obtain a waveform with the same periodicity as the HR from each SCG signal. The peaks of this waveform were then detected, considering a minimum peak distance and minimum peak prominence of 60 × *f*_*s*_/*HR*_*θ*_ and 0.2 × *SD*_*SCG*_, respectively, where *HR*_*θ*_ and *SD*_*SCG*_ represent a maximum HR threshold and the standard deviation of the SCG signal. In this study, *HR*_*θ*_ was set to 120 bpm. The instantaneous HR from each SCG signal was then calculated from the inter-peak intervals. Finally, the average HR was calculated from the instantaneous HRs of all SCG signals.

Note that the value of the minimum peak distance, i.e., 60 × *f*_*s*_/*HR*_*θ*_, was constant regardless of the SCG signal and subject’s cardiac cycle duration. As a result, instances occurred where the algorithm either missed peaks or incorrectly identified additional peaks, leading to less accurate HR estimations. However, despite this limitation, our algorithm provided accurate HR estimations for the majority of signals in both right-to-left and head-to-foot SCG grids for each subject. Using this to the advantage of our algorithm, in the second step of our algorithm, we utilized all the average HRs estimated in the first step for each subject to improve the accuracy of the peak detection. Specifically, the algorithm assessed the standard deviation of the average HRs derived from all SCG signals of each subject. In cases where the standard deviation surpassed a predefined threshold, indicating variability in average HRs, the algorithm dynamically calculated a personalized minimum peak distance for that subject. This adaptive approach aimed to refine peak detection, ultimately improving the precision of the estimated HR.

To determine the personalized minimum peak distance, the collection of average HRs estimated from all the SCG signals in the right-to-left and head-to-foot direction were clustered into two clusters using k-means clustering. Assuming the majority of HRs estimated in the first step were accurate, the larger cluster inherently provided a more reliable representation of the HR. To enhance the cluster’s integrity, we eliminated outliers by excluding average HRs outside the 95% confidence interval of values within the larger cluster. We then calculated the mean of the remaining average HRs in the augmented cluster and added an offset value of 20 bpm to it. This number (*HR*_*p*_) was used to calculate the personalized minimum peak distance as 60 × *f*_*s*_/*HR*_*p*_. Consequently, we recalculated all instantaneous HRs using this refined minimum peak distance. Finally, outliers outside of the 95% confidence interval were removed from the instantaneous HR to calculate the average HR for every SCG signal in the right-to-left and head-to-foot SCG grids.

## Supplementary information

Supplementary File

Supplementary Video 1

## Data Availability

The data generated and analyzed during the current study are not publicly available due to the IRB requirements, but a part of the processed data may be available from the corresponding author on a reasonable request.
