# Supplementary material for: From video to vital signs: a new method for contactless multichannel seismocardiography
Source: NPJ Cardiovasc Health. 2025 Jan 10;2:1. doi: 10.1038/s44325-024-00034-6 (PMC12810757; doi:10.1038/s44325-024-00034-6)
Supplement: Supplementary file 3 — Supplementary File [file 44325_2024_34_MOESM1_ESM.pdf]

Supplementary Materials for ”From Video to Vital Signs: A New Method for Contactless Multichannel Seismocardiography”

Mohammad Muntasir Rahman[1], Bahram Kakavand[2,3], William Van Wurm[4], William L. Holman[5], Mohammad Reza Movahed[6,7], and Amirtahà Taebi[1,*]

[1]Department of Agricultural and Biological Engineering, Mississippi State University, Mississippi State, MS 39762, USA
[2]Department of Pediatrics, Division of Pediatric Cardiology, Nemours Children's Hospital, Orlando, FL 32827, USA
[3]College of Medicine, University of Central Florida, Orlando, FL 32827, USA
[4]Physician Assistant Studies Program, Mississippi State University, Meridian, MS 39301, USA
[5]Department of Surgery, Division of Cardiothoracic Surgery, University of Alabama at Birmingham, Birmingham, AL 35233, USA
[6]Sarver Heart Center, University of Arizona, Tucson, AZ 85724, USA
[7]College of Medicine, University of Arizona, Phoenix, AZ 85004, USA
[*]Corresponding author: ataebi@abe.msstate.edu

Seismocardiography (SCG) is a technique that non-invasively measures the chest wall's local vibrations caused by the heart's mechanical activity. Traditionally, SCG signals have been recorded using accelerometers placed at a single location on the chest wall. This study presents an innovative, cost-effective SCG method that utilizes standard smartphone videos to capture data from multiple chest locations. The analysis of vibrations from multiple points can offer a more thorough understanding of the heart's mechanical activity compared to signals obtained solely from a single chest location. Our approach employs computer vision and deep learning techniques to extract and improve the resolution of multichannel SCG maps obtained by video capture of chest movement. We attached a grid of patterned stickers to the chest surface and recorded videos of chest movements during different respiratory phases. Using a deep learning-based object detector and a template tracking method, we tracked the stickers across video frames and extracted the corresponding SCG signals from sticker displacements. We also developed a robust algorithm to estimate heart rate (HR) from these chest videos and identify the optimal chest location for HR estimation. The method was tested on 28 chest videos captured from 14 healthy participants. The results demonstrated that our method effectively extracted multichannel SCG maps and enhanced their resolution with a mean squared error of 0.1078 and 0.0418 for right-to-left and head-to-foot SCG signals, respectively. We observed intersubject chest vibration patterns corresponding to cardiac events including opening and closure of the heart valves. Moreover, our algorithm accurately estimated HR from 1,968 SCG signals extracted from the videos compared to the gold-standard HR measured from each subject's electrocardiogram (bias ± 1.96 SD $= 0.04 \pm 2.14$ bpm; $r = 0.99, p < 0.001$). The findings from this study underscore the potential of our approach in developing a cardiac monitoring tool using a smartphone. Such an approach would be widely accessible to the general public and might provide more timely detection of diseases.

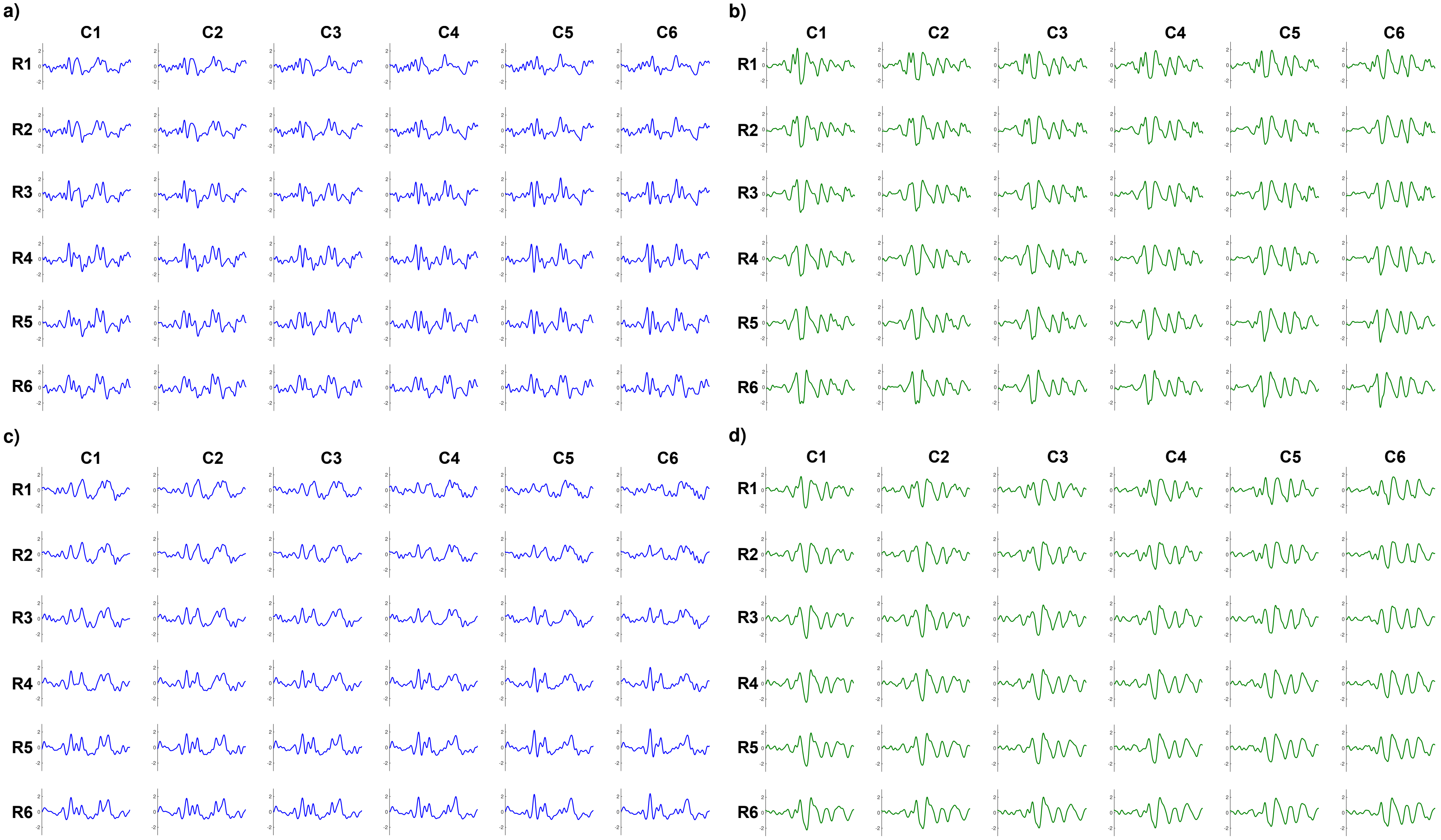

Supplementary Fig. 1. Vision-based SCG signal for Subject 01. **a)** Right-to-left chest vibration at the end of exhalation. **b)** Head-to-foot chest vibration at the end of exhalation. **c)** Right-to-left chest vibration at the end of inhalation. **d)** Head-to-foot chest vibration at the end of inhalation.

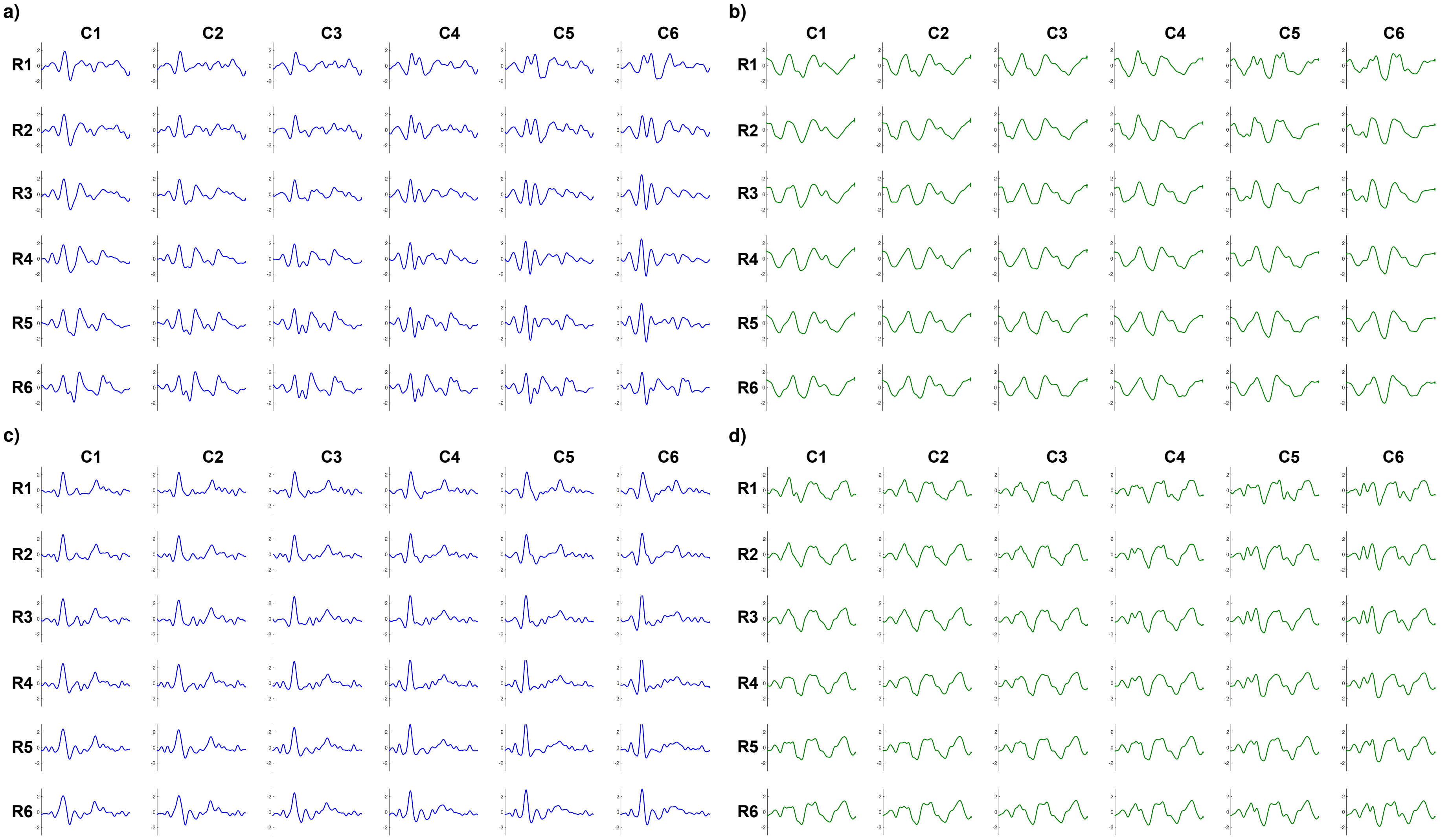

Supplementary Fig. 2. Vision-based SCG signal for Subject 02. **a)** Right-to-left chest vibration at the end of exhalation. **b)** Head-to-foot chest vibration at the end of exhalation. **c)** Right-to-left chest vibration at the end of inhalation. **d)** Head-to-foot chest vibration at the end of inhalation.

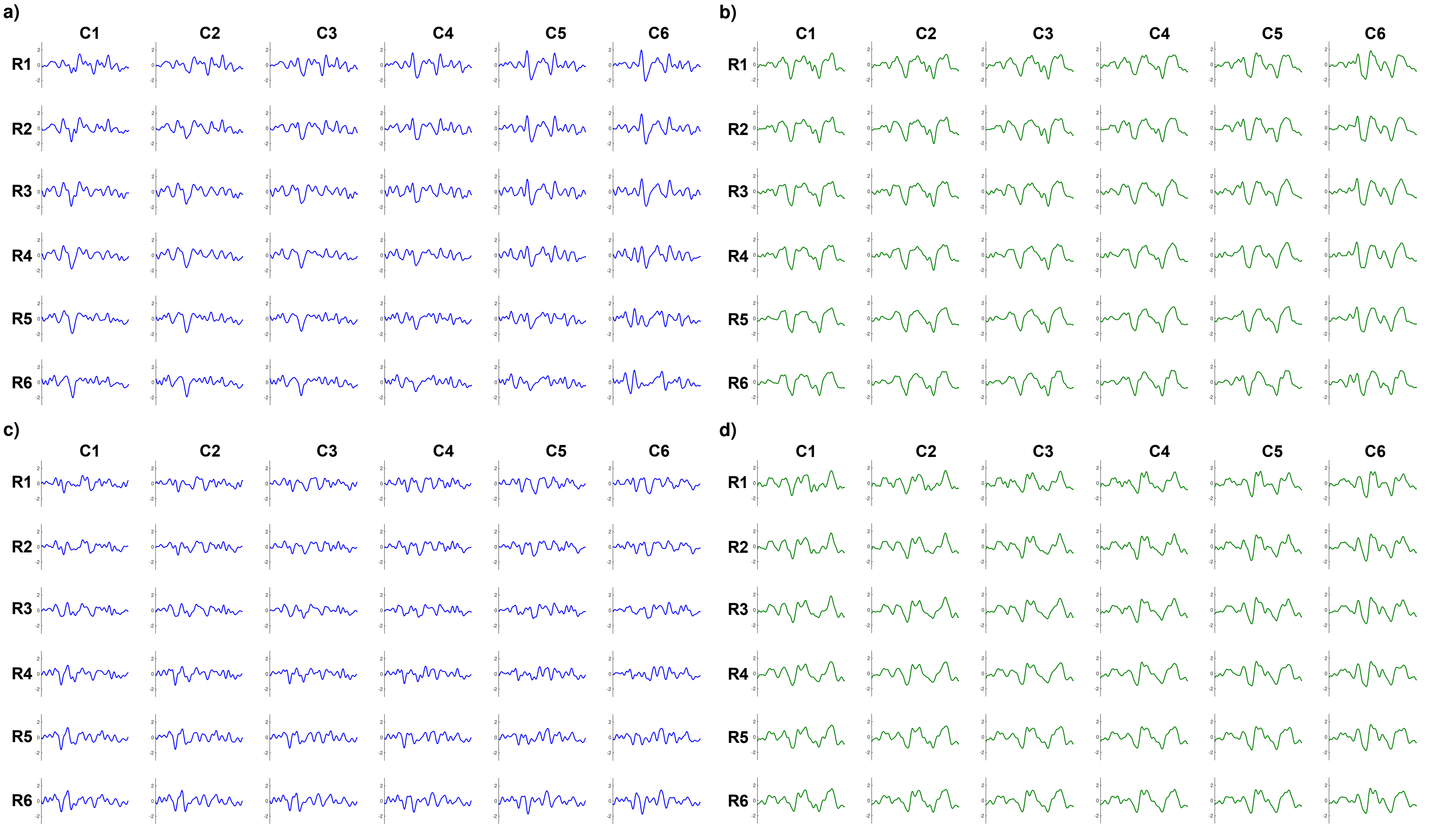

Supplementary Fig. 3. Vision-based SCG signal for Subject 03. **a)** Right-to-left chest vibration at the end of exhalation. **b)** Head-to-foot chest vibration at the end of exhalation. **c)** Right-to-left chest vibration at the end of inhalation. **d)** Head-to-foot chest vibration at the end of inhalation.

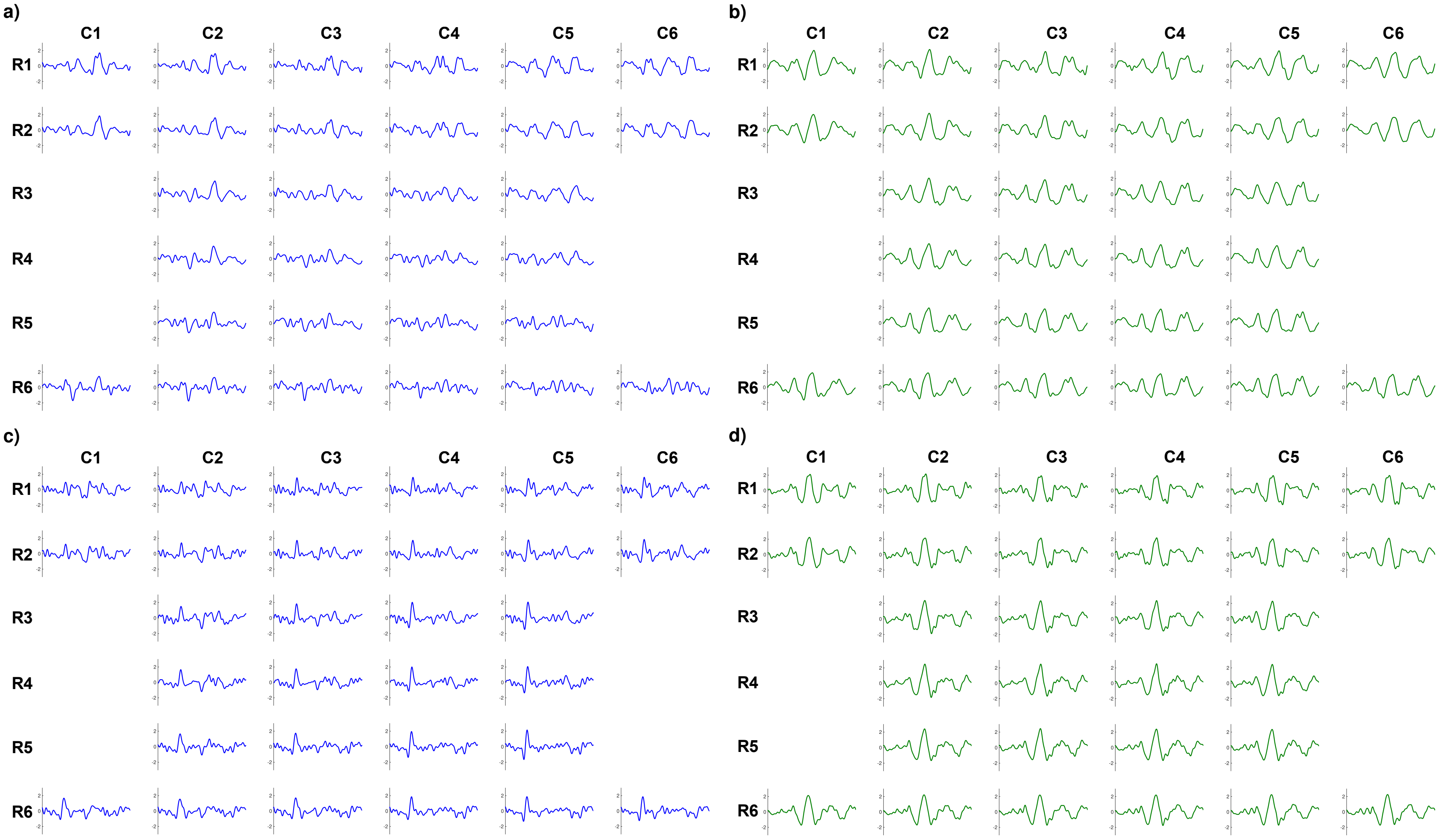

Supplementary Fig. 4. Vision-based SCG signal for Subject 04. **a)** Right-to-left chest vibration at the end of exhalation. **b)** Head-to-foot chest vibration at the end of exhalation. **c)** Right-to-left chest vibration at the end of inhalation. **d)** Head-to-foot chest vibration at the end of inhalation.

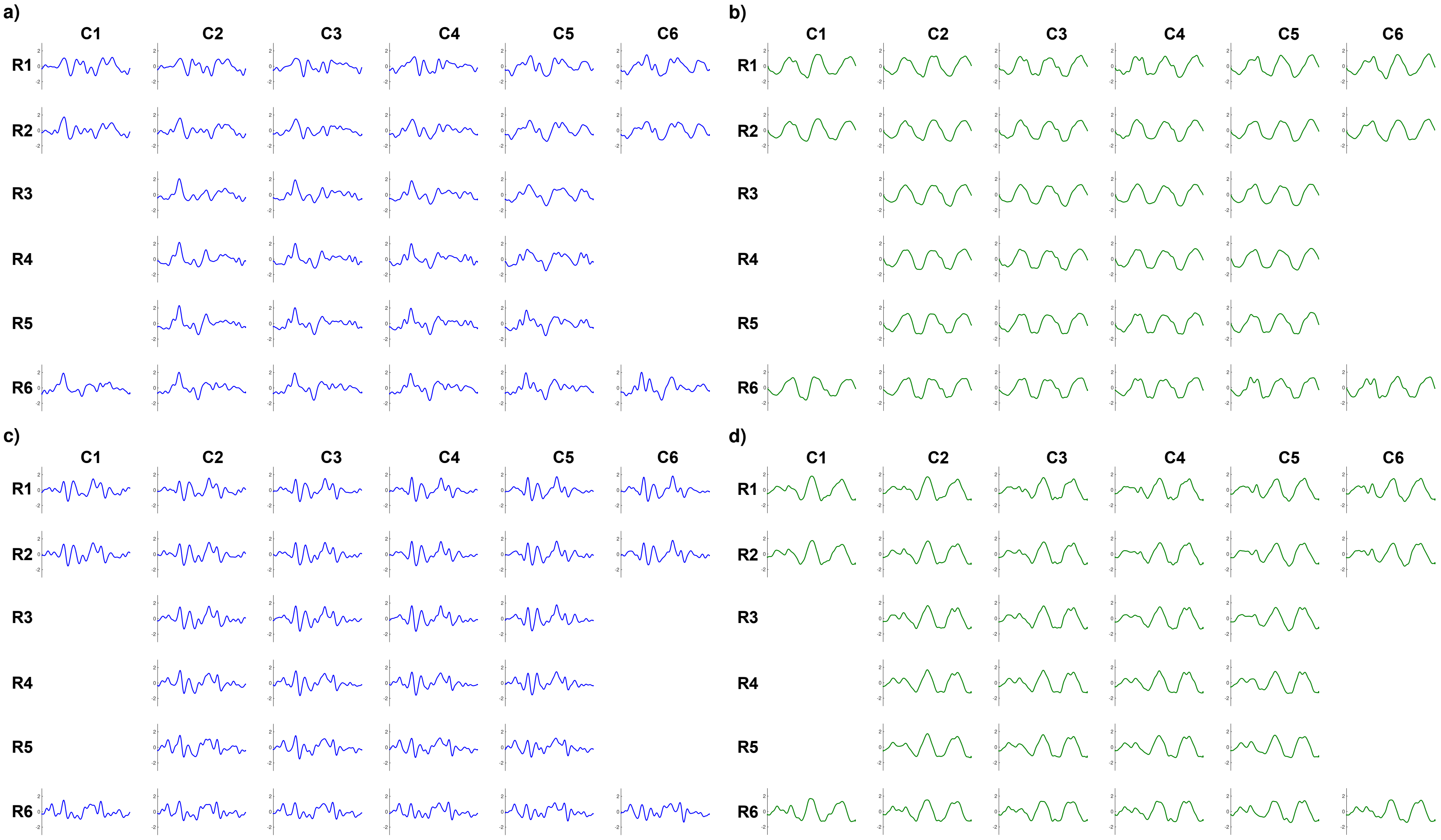

Supplementary Fig. 5. Vision-based SCG signal for Subject 05. **a)** Right-to-left chest vibration at the end of exhalation. **b)** Head-to-foot chest vibration at the end of exhalation. **c)** Right-to-left chest vibration at the end of inhalation. **d)** Head-to-foot chest vibration at the end of inhalation.

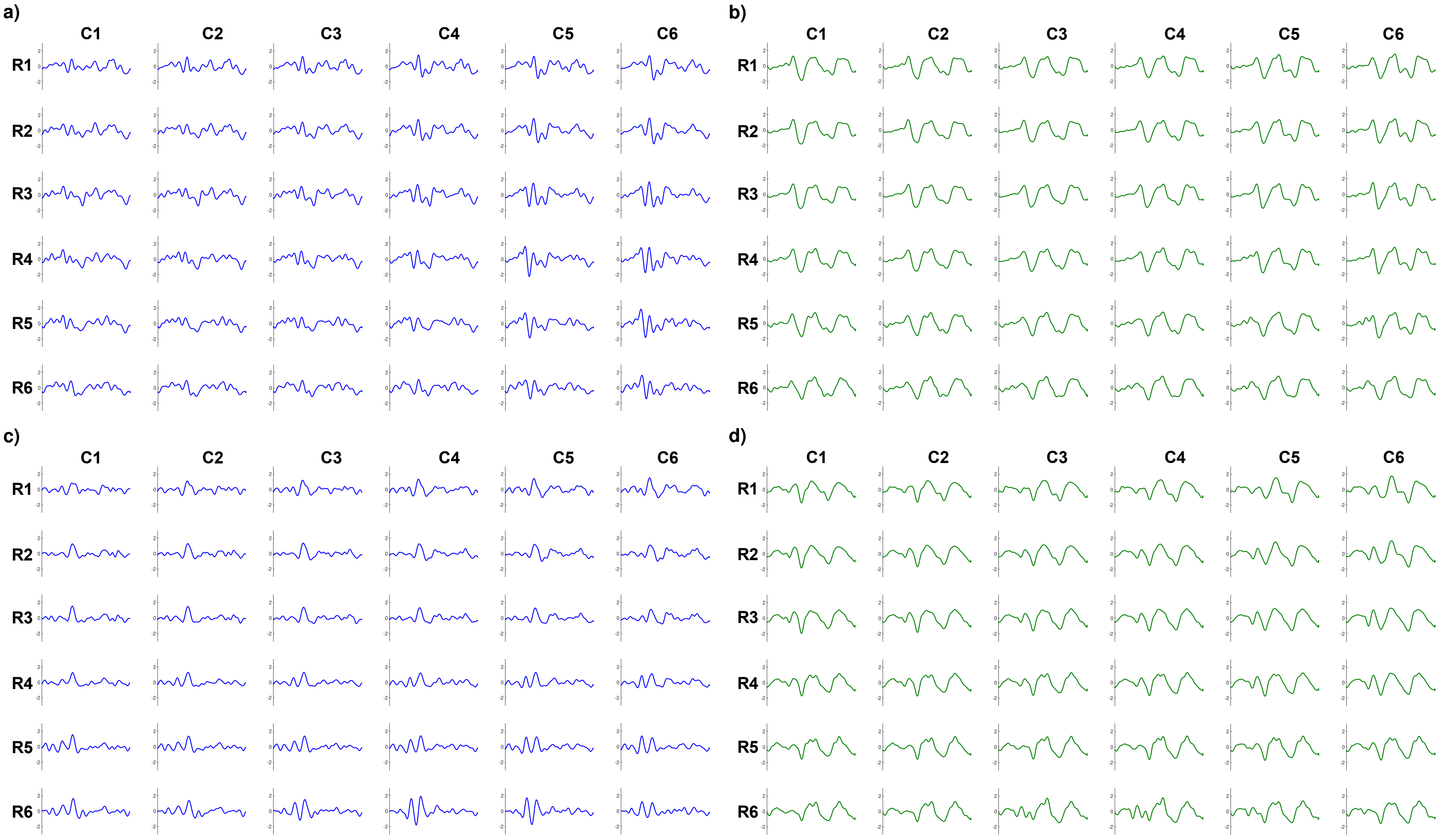

Supplementary Fig. 6. Vision-based SCG signal for Subject 06. **a)** Right-to-left chest vibration at the end of exhalation. **b)** Head-to-foot chest vibration at the end of exhalation. **c)** Right-to-left chest vibration at the end of inhalation. **d)** Head-to-foot chest vibration at the end of inhalation.

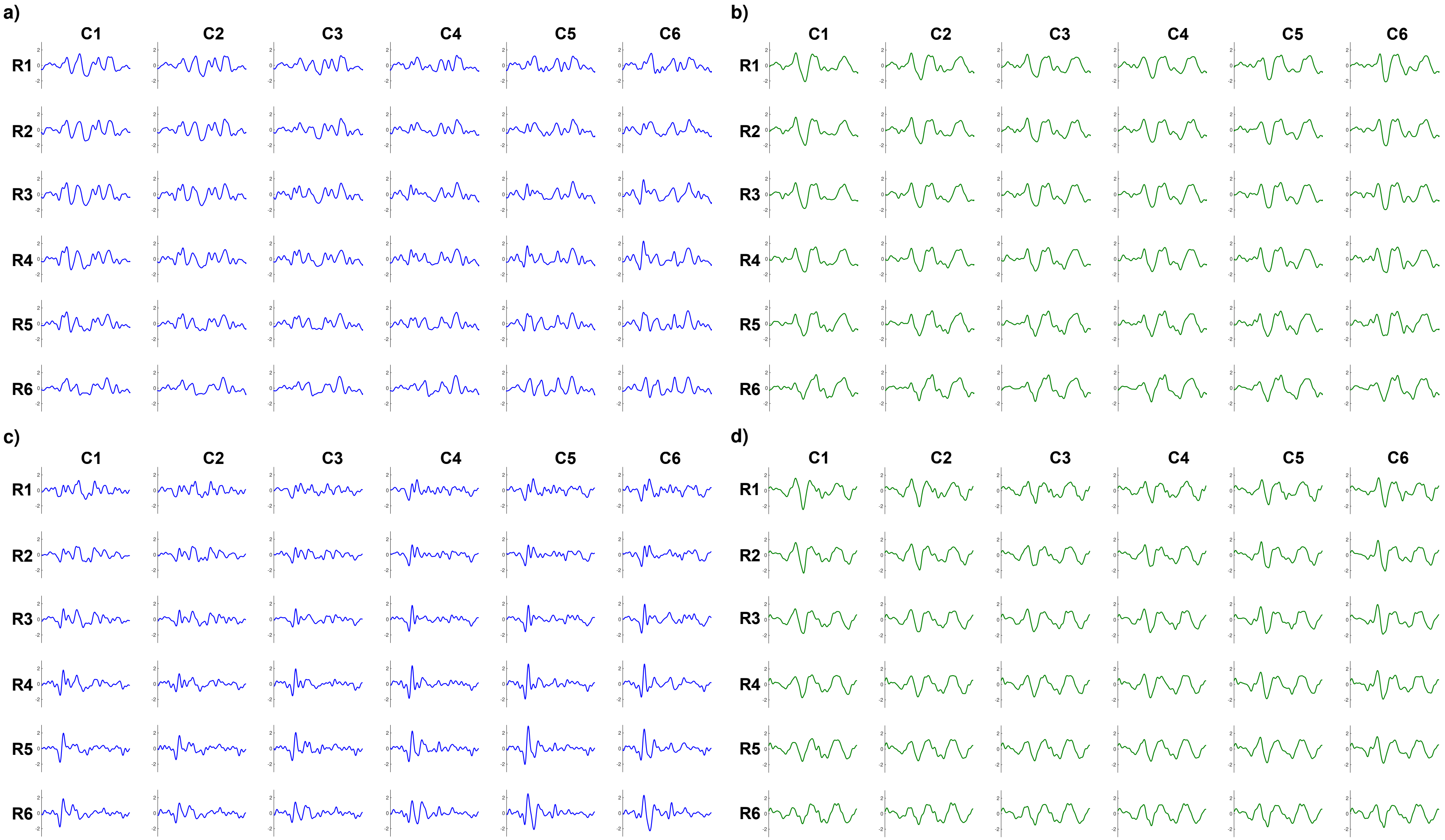

Supplementary Fig. 7. Vision-based SCG signal for Subject 07. **a)** Right-to-left chest vibration at the end of exhalation. **b)** Head-to-foot chest vibration at the end of exhalation. **c)** Right-to-left chest vibration at the end of inhalation. **d)** Head-to-foot chest vibration at the end of inhalation.

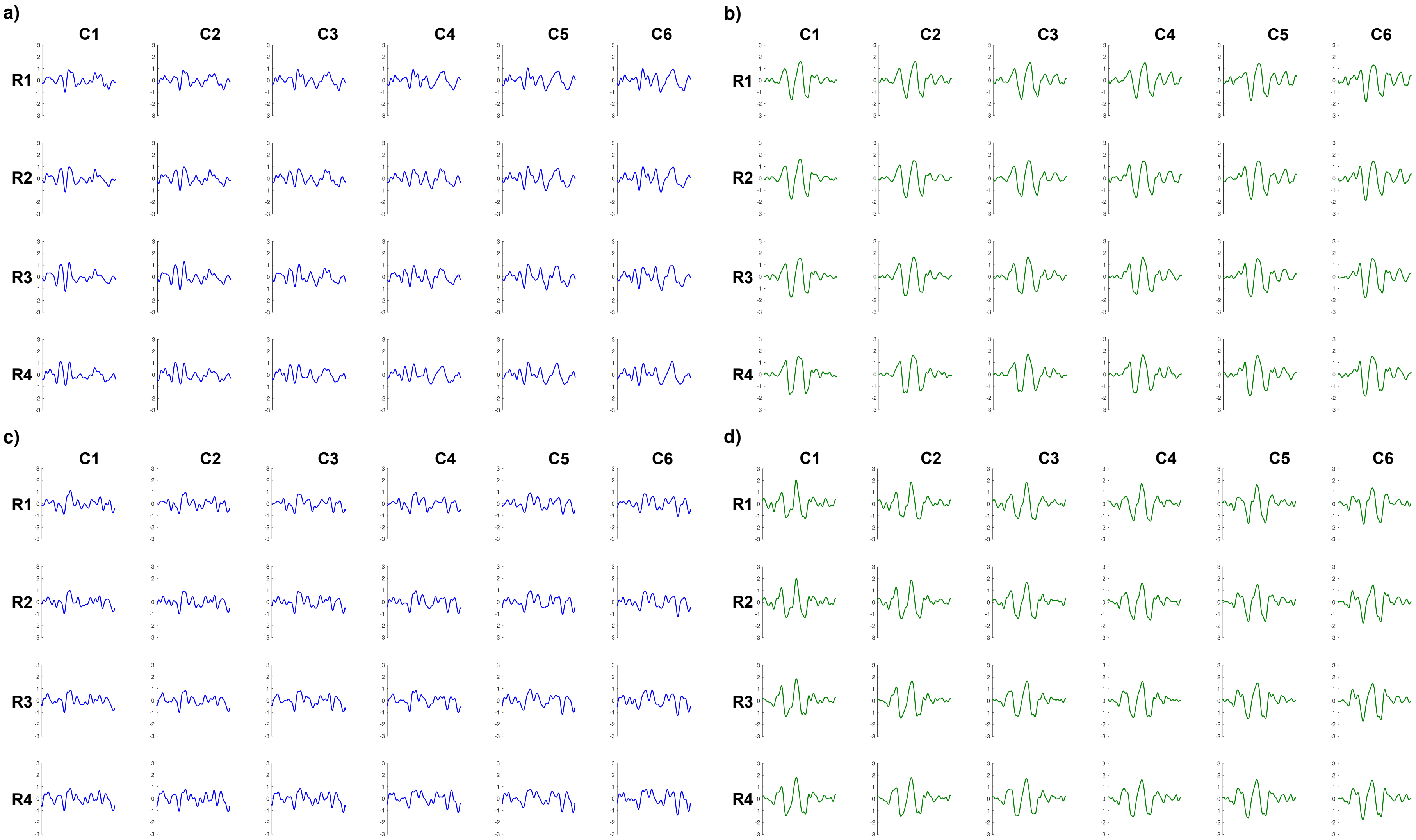

Supplementary Fig. 8. Vision-based SCG signal for Subject 08. **a)** Right-to-left chest vibration at the end of exhalation. **b)** Head-to-foot chest vibration at the end of exhalation. **c)** Right-to-left chest vibration at the end of inhalation. **d)** Head-to-foot chest vibration at the end of inhalation.

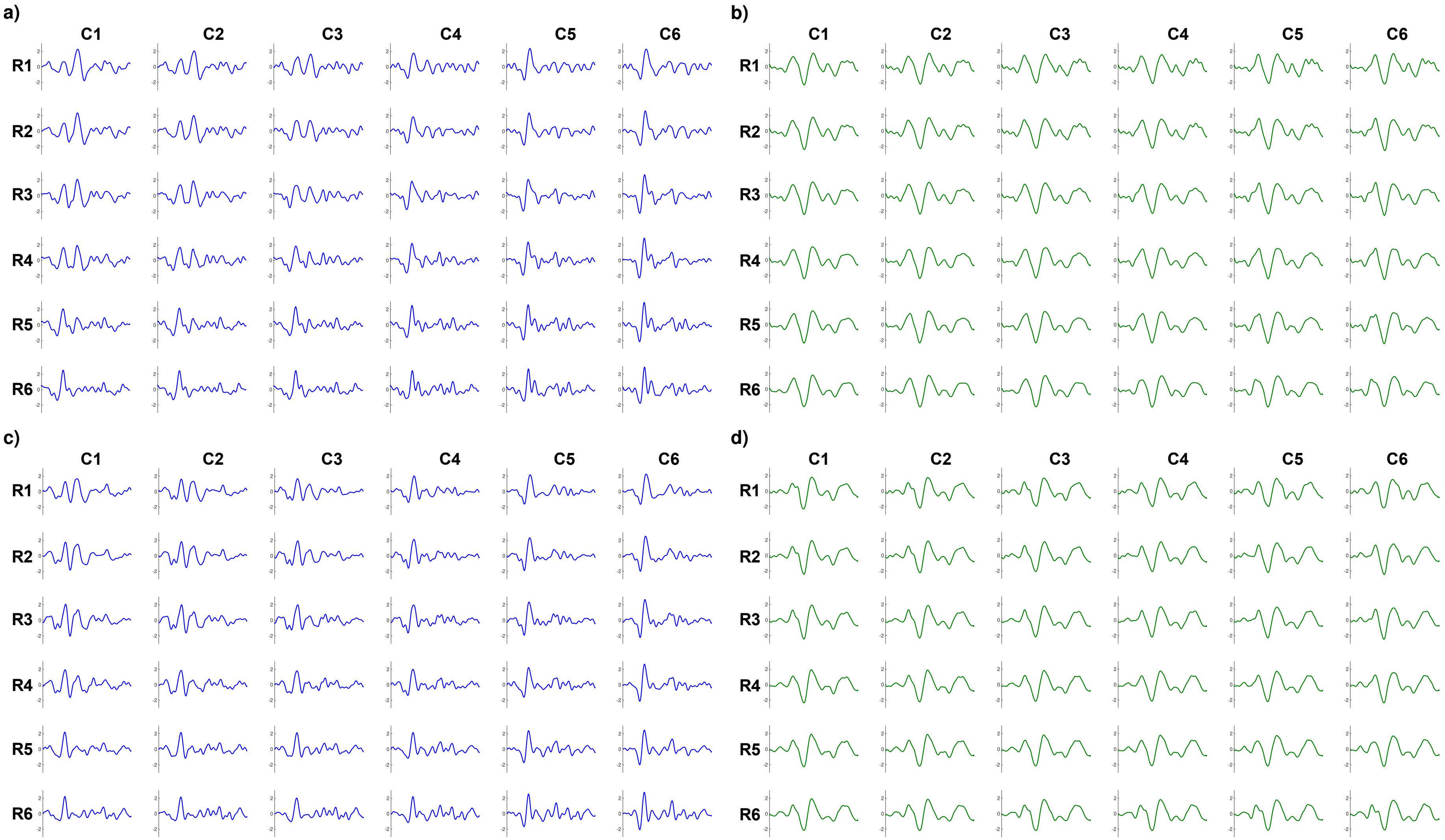

Supplementary Fig. 9. Vision-based SCG signal for Subject 09. **a)** Right-to-left chest vibration at the end of exhalation. **b)** Head-to-foot chest vibration at the end of exhalation. **c)** Right-to-left chest vibration at the end of inhalation. **d)** Head-to-foot chest vibration at the end of inhalation.

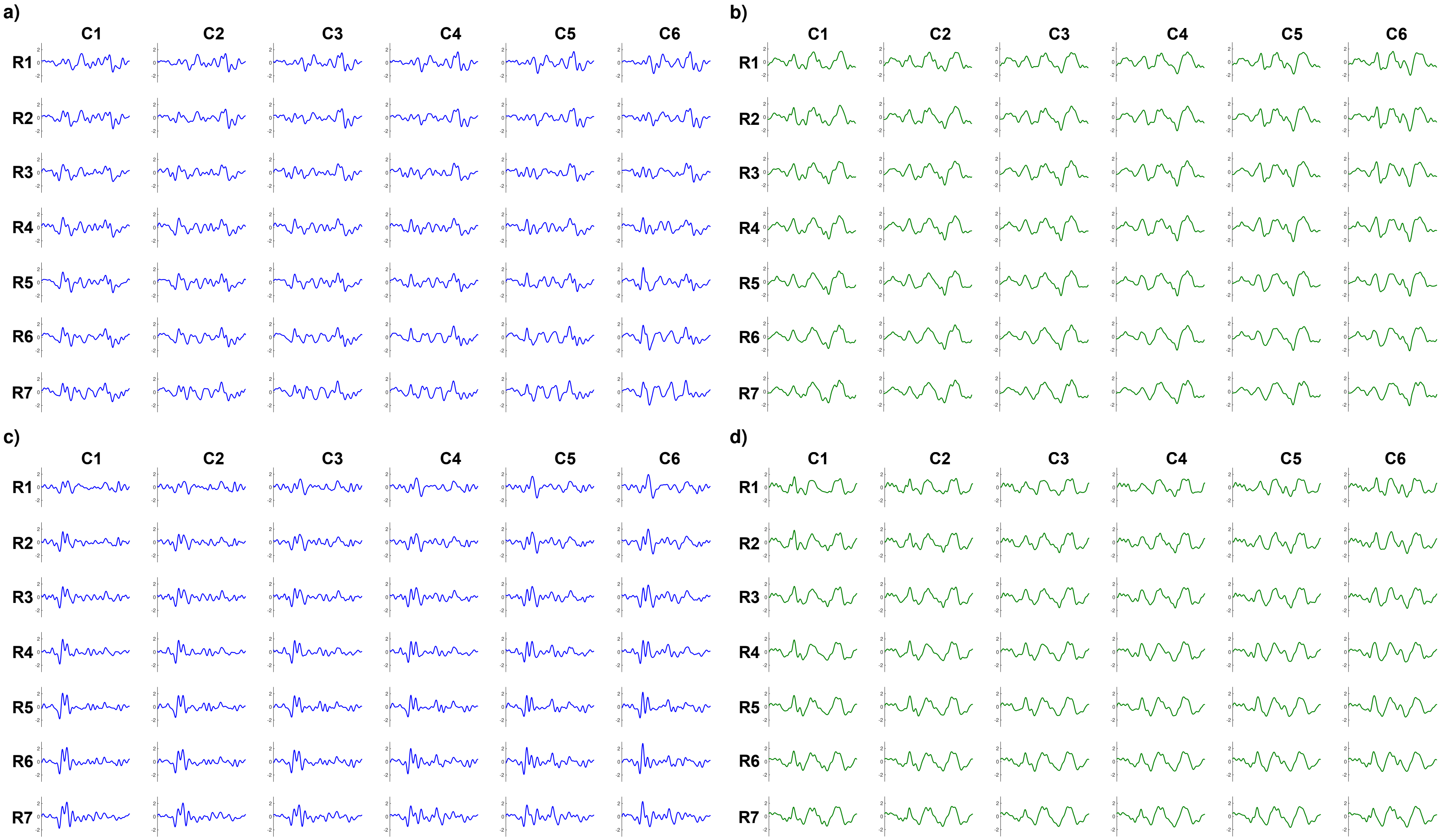

Supplementary Fig. 10. Vision-based SCG signal for Subject 10. **a)** Right-to-left chest vibration at the end of exhalation. **b)** Head-to-foot chest vibration at the end of exhalation. **c)** Right-to-left chest vibration at the end of inhalation. **d)** Head-to-foot chest vibration at the end of inhalation.

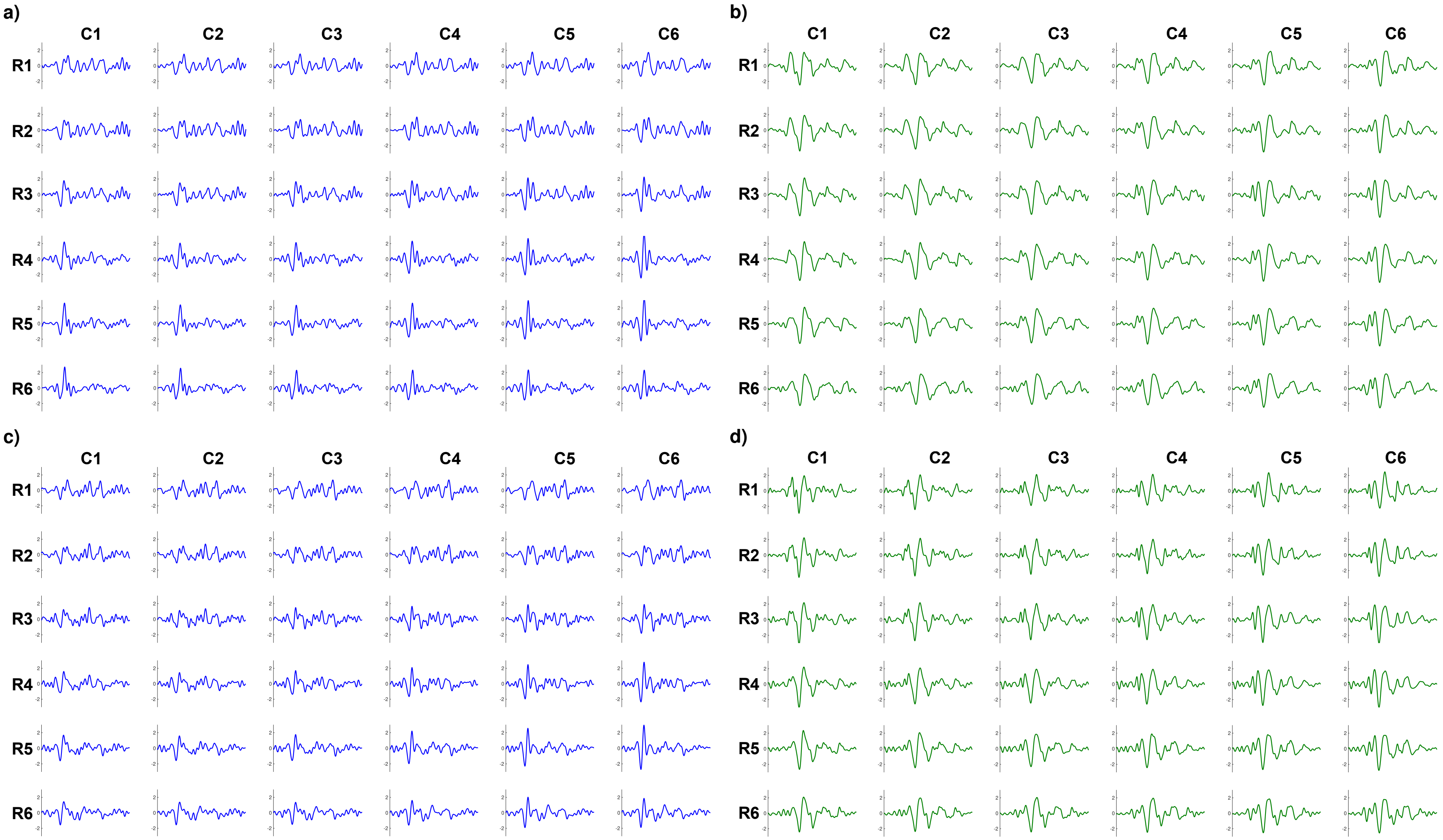

Supplementary Fig. 11. Vision-based SCG signal for Subject 11. **a)** Right-to-left chest vibration at the end of exhalation. **b)** Head-to-foot chest vibration at the end of exhalation. **c)** Right-to-left chest vibration at the end of inhalation. **d)** Head-to-foot chest vibration at the end of inhalation.

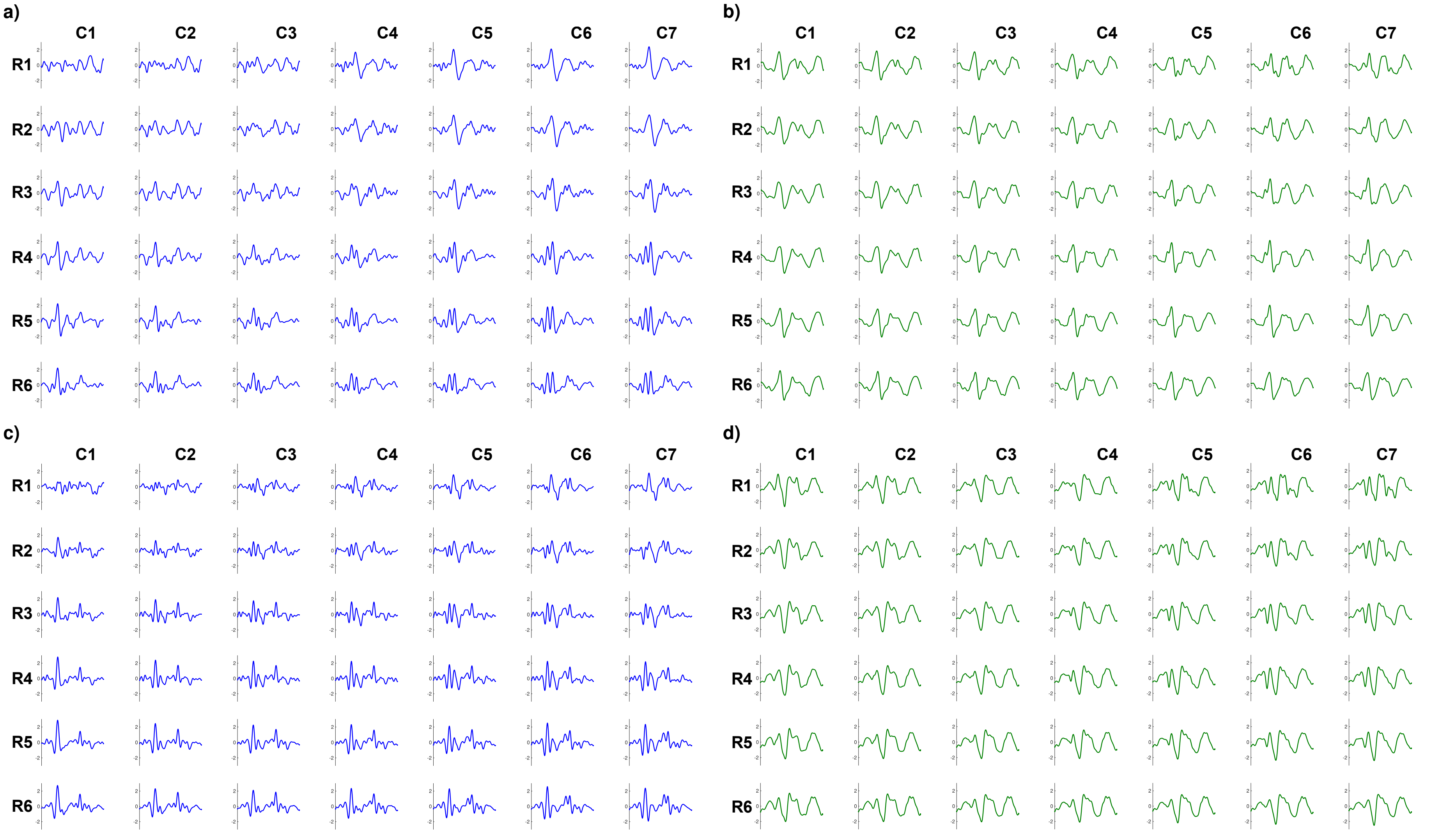

Supplementary Fig. 12. Vision-based SCG signal for Subject 12. **a)** Right-to-left chest vibration at the end of exhalation. **b)** Head-to-foot chest vibration at the end of exhalation. **c)** Right-to-left chest vibration at the end of inhalation. **d)** Head-to-foot chest vibration at the end of inhalation.

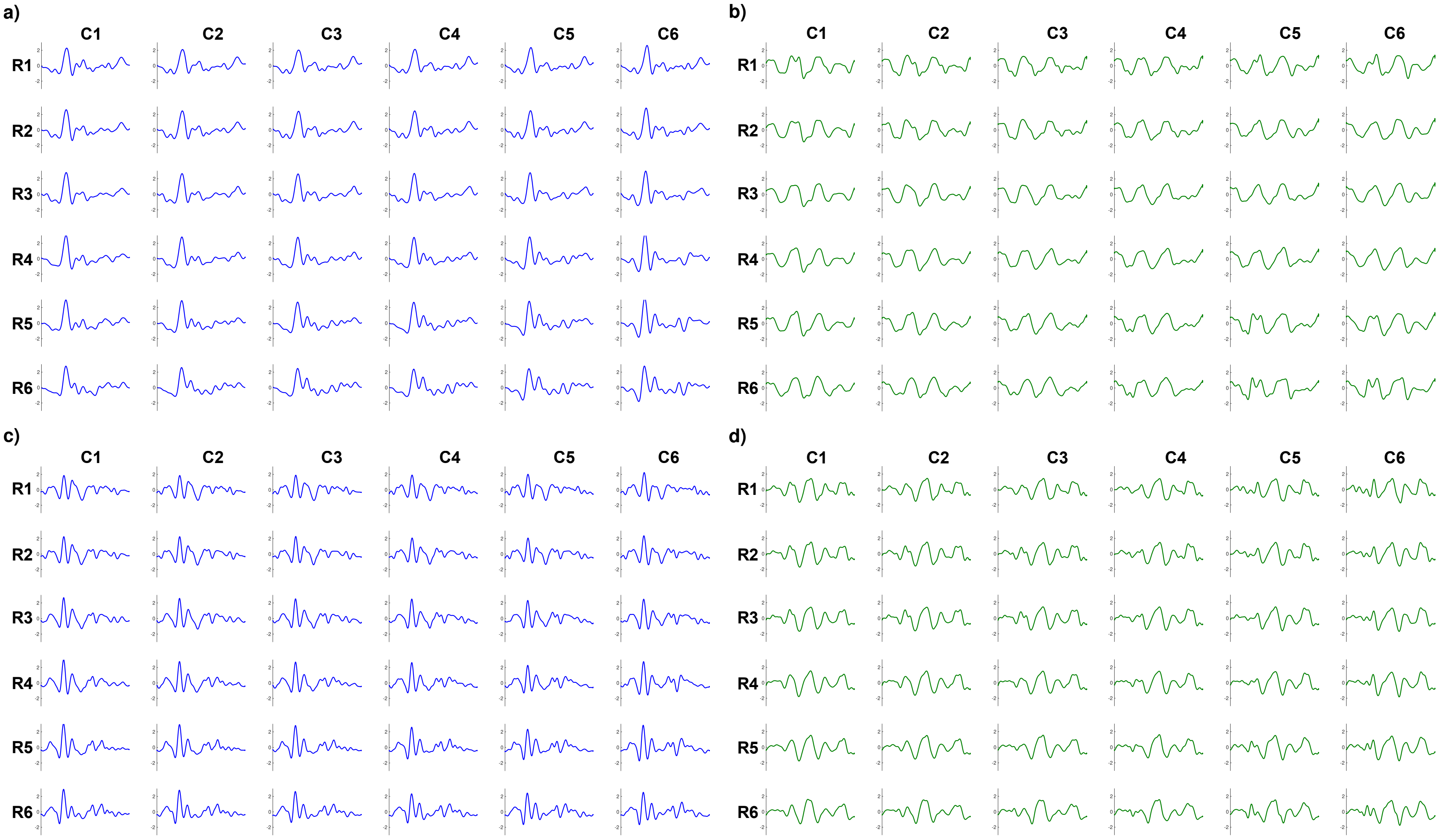

Supplementary Fig. 13. Vision-based SCG signal for Subject 13. **a)** Right-to-left chest vibration at the end of exhalation. **b)** Head-to-foot chest vibration at the end of exhalation. **c)** Right-to-left chest vibration at the end of inhalation. **d)** Head-to-foot chest vibration at the end of inhalation.

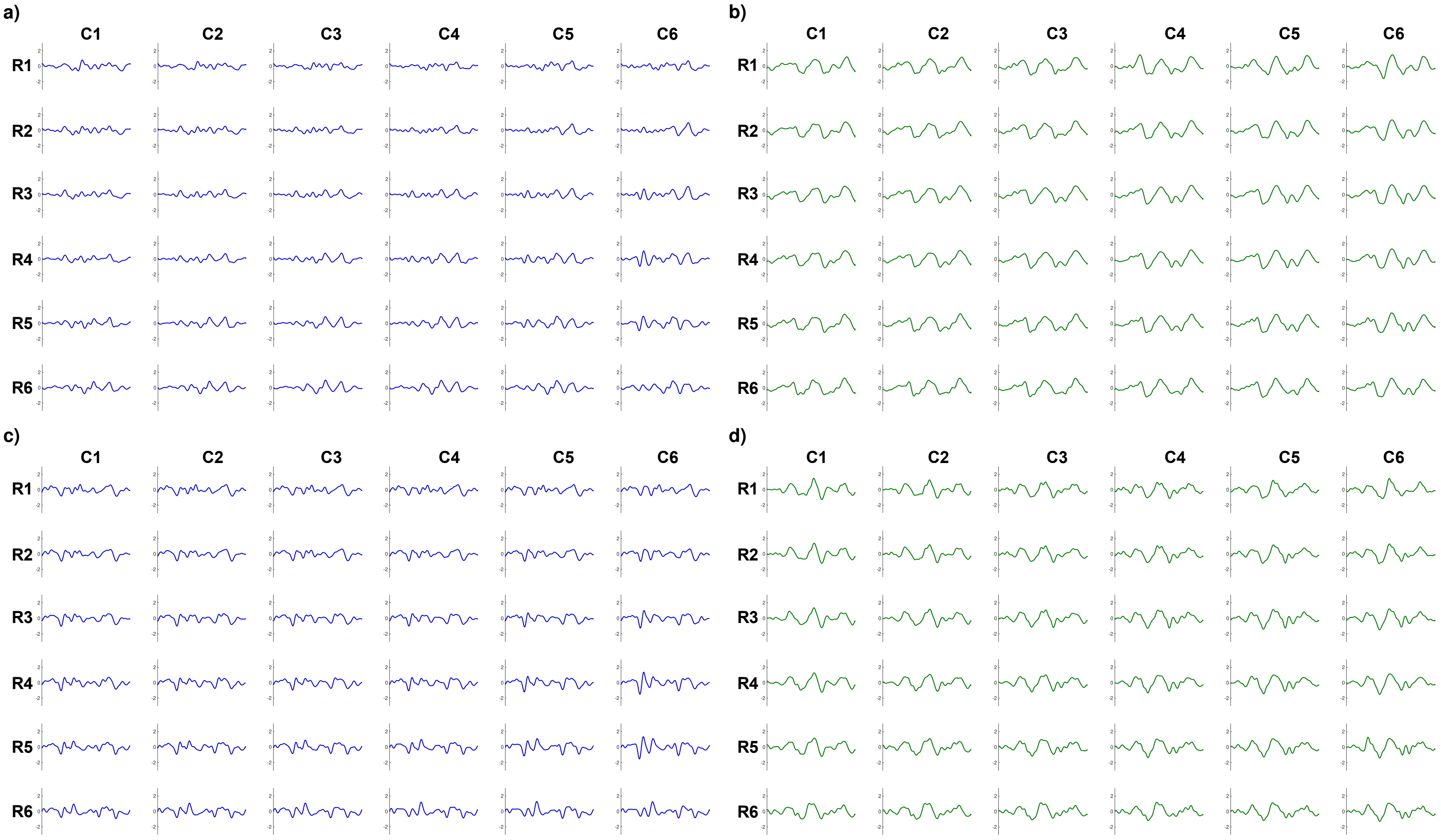

Supplementary Fig. 14. Vision-based SCG signal for Subject 14. **a)** Right-to-left chest vibration at the end of exhalation. **b)** Head-to-foot chest vibration at the end of exhalation. **c)** Right-to-left chest vibration at the end of inhalation. **d)** Head-to-foot chest vibration at the end of inhalation.

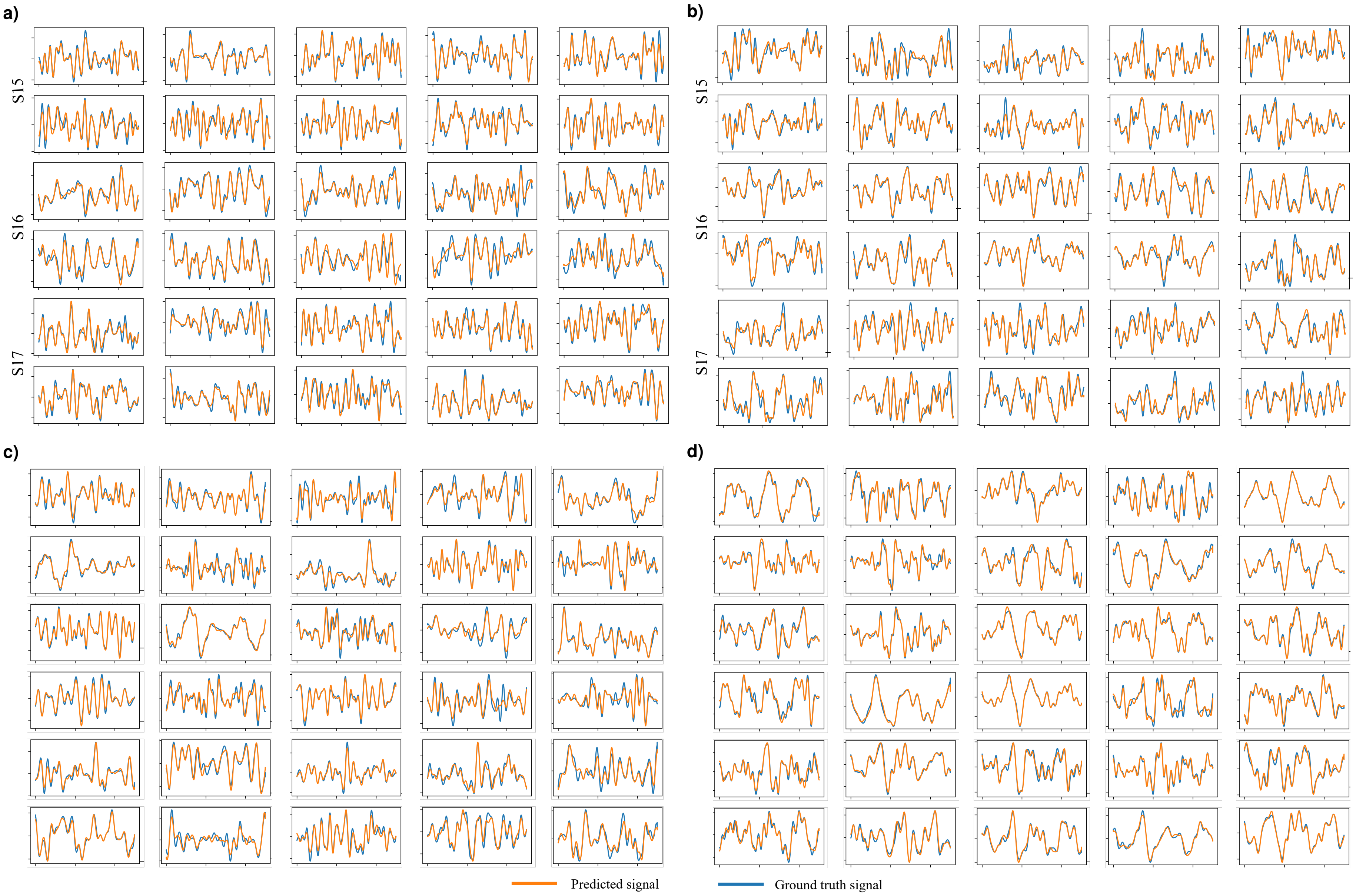

Supplementary Fig. 15. Samples of predicted signals from the CNN models. **a)** Samples from the test set of the right-to-left model. **b)** Samples from the test set of the head-to-foot model. **c)** Samples from the validation set of the right-to-left model. **d)** Samples from the validation set of the head-to-foot model.

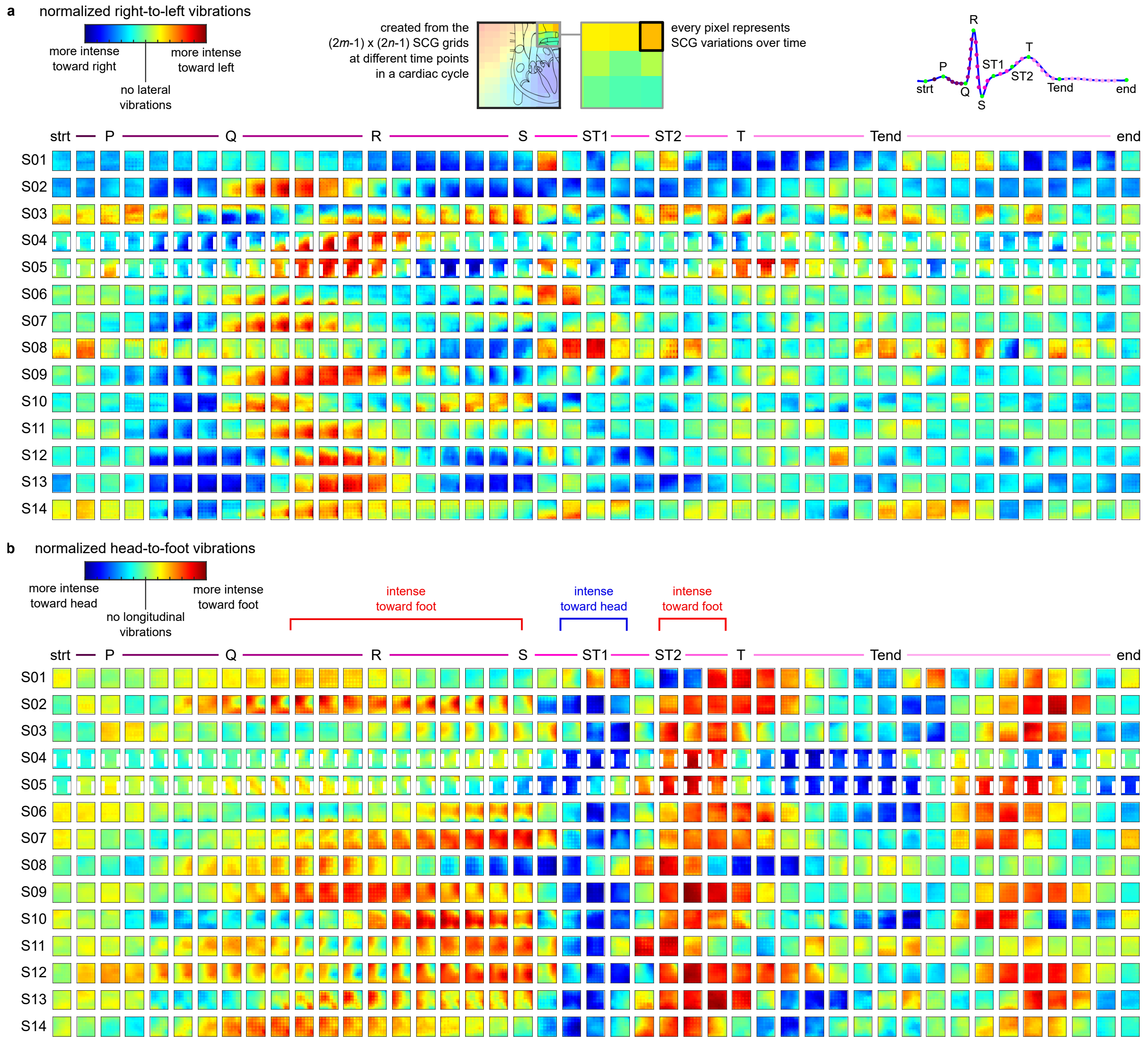

Supplementary Fig. 16. Enhanced resolution SCG maps at the end of inhalation. **a)** Normalized right-to-left chest vibrations, and **b)** Normalized head-to-foot chest vibrations for all 14 subjects during breath-hold at the end of inhalation. Each pixel of the maps shows the temporal variations of the corresponding ensemble average SCG signal during a cardiac cycle. Time-varying vibration maps were created using the enhanced-resolution $(2m-1)\times(2n-1)$ SCG grids and aligned with respect to the ECG reference points of each subject for better visualization of the results. However, it is noteworthy that the time step between the maps and the timing of maps corresponding to the same ECG reference point for different subjects are not uniform, and depend on the cardiac cycle duration of each subject.

Supplementary Video Captions

Supplementary Video. 1. SCG signal in the head-to-foot direction, SCG_{hf} (yellow signal), extracted from the sticker shown by the yellow rectangle. The ECG signal is shown in green color. Instantaneous heart rates extracted from these signals are shown on the right side. At the end of the video, the average heart rate (mean $\pm$ standard deviation) is shown for each method. Consent was obtained for publishing the subject's chest video.
